# Immune biomarker landscape and fusion partner–phenotype associations in thoracic and head-and-neck NUT carcinoma

**DOI:** 10.3389/fimmu.2026.1834366

**Published:** 2026-05-28

**Authors:** Shuang Xiang, Zhuomiao Ye, Yifan Dai, Shipeng Shang, Dan Yang, Tao Cheng, Chao Deng, Minghui Zhang, Mingzhu Yin

**Affiliations:** 1 Medical Pathology Center (MPC), Chongqing University Three Gorges Hospital, Chongqing University, Wanzhou, Chongqing, China; 2 Clinical Research Center (CRC), Chongqing University Three Gorges Hospital, Chongqing University, Wanzhou, Chongqing, China; 3 Chongqing University Three Gorges Hospital and Academy for Advanced Interdisciplinary Technology, CQU-Ferenc Krausz Nobel Laureate Scientific Workstation, Wanzhou, Chongqing, China; 4 Cancer Early Detection and Treatment Center (CEDTC), Chongqing University Three Gorges Hospital, Chongqing University, Wanzhou, Chongqing, China; 5 Department of Breast Surgery, Chongqing University Three Gorges Hospital, Chongqing University, Wanzhou, Chongqing, China

**Keywords:** bibliometrics, head and neck, NUT carcinoma, NUTM1 fusion, PD-L1, thorax

## Abstract

**Background:**

Thoracic and head and neck NUT carcinoma is a rare, highly aggressive NUTM1-rearranged malignancy with substantial diagnostic difficulty, marked phenotypic heterogeneity, and no established biomarker framework for immunotherapy stratification. Although fusion partners may contribute to clinicopathologic diversity, their relationship to phenotype and immune biomarkers remains insufficiently defined.

**Materials and methods:**

We performed an integrated literature-based analysis of thoracic and head and neck NUT carcinoma using English-language publications indexed in the Web of Science Core Collection from 1991 to 2025. Eligible studies were screened for clinicopathologic, fusion, and immune-biomarker data. Case-level variables were harmonized into anatomic and lineage-based strata to evaluate associations between fusion partners, phenotype, and immune features. PD-L1 status was harmonized into three descriptive categories based on author interpretation and/or reported TPS/CPS thresholds, and a binary sensitivity framework, Negative versus Any expression, was additionally applied to reduce the impact of heterogeneous assays and scoring systems. MSI and TMB were also extracted when available. Associations were assessed using two-sided Fisher’s exact test with odds ratios and 95% confidence intervals. To strengthen the robustness and translational relevance of the findings, we additionally analyzed an independent retrospective institutional cohort of thoracic and head and neck NUT carcinoma.

**Results:**

The integrated clinicopathologic dataset included 229 thoracic/head and neck cases with definitive fusion annotations from 109 studies. Squamous lineage predominated (71.62%), but non-squamous and mixed phenotypes were also identified. *BRD4::NUTM1* was significantly enriched in squamous lineage and thoracic squamous subsets, whereas *YAP1::NUTM1* and *MGA::NUTM1* were preferentially associated with non-squamous phenotypes and specific strata. In the immune-biomarker subset, PD-L1 was predominantly negative (76.92%), with high expression being uncommon (6.41%). Thoracic squamous tumors showed significant enrichment for PD-L1 negativity. Among cases with both fusion and PD-L1 data, a binary sensitivity analysis confirmed that PD-L1 negativity predominated across fusion-defined subsets, with no significant association between fusion partner and PD-L1 status. Across reported MSI/TMB cases, tumors were uniformly MSS and generally low in TMB. An independent institutional cohort of 59 patients partially corroborated the major literature-derived signals, particularly *BRD4::NUTM1* predominance among fusion-annotated cases and PD-L1 negativity among tested tumors, with no significant difference from the literature-derived pooled estimates.

**Conclusion:**

This study defines the immune biomarker landscape of thoracic and head and neck NUT carcinoma within a clinicopathologic and fusion-partner framework. The predominance of PD-L1 negativity together with MSS/low-TMB features suggests a generally immunologically “cold” phenotype in most reported tumors, while exploratory fusion partner–specific enrichment patterns may help refine diagnostic suspicion and generate hypotheses for future biomarker-guided stratification. These findings provide a translational basis for rare-cancer immunobiology research and for the rational design of biomarker-integrated prospective studies.

## 1 Introduction

NUT carcinoma is a rare yet highly aggressive malignancy driven by *NUTM1* gene rearrangements. The prototypical t(15;19)-associated aggressive carcinoma was first reported in 1991 (1). Early reports emphasized tumors arising in midline structures, leading to the historical term “NUT midline carcinoma.” The entity subsequently gained broader recognition in WHO classification frameworks around 2015 onward, including thoracic pathology contexts (2). Importantly, *NUTM1* rearrangement is not exclusive to NUT carcinoma and can also occur in other neoplastic contexts, including round cell sarcomas, acute leukemia, and selected adnexal tumors, underscoring the need for careful clinicopathologic correlation and molecular confirmation (3). In routine practice, NUT carcinoma often presents as a rapidly progressive poorly differentiated or undifferentiated malignancy with limited radiologic and morphologic specificity, and it is frequently misclassified as poorly differentiated squamous cell carcinoma, undifferentiated carcinoma, or other small round cell/sarcomatoid tumors (4, 5). Definitive diagnosis typically relies on NUT immunohistochemistry (NUT-IHC) demonstrating nuclear positivity, followed by confirmatory testing with FISH or NGS to document *NUTM1* rearrangement. Owing to disease rarity, limited awareness, and marked morphologic heterogeneity, NUT-IHC is not consistently incorporated into standard diagnostic workflows, contributing to under-recognition and delayed confirmation (6, 7). Therapeutically, no universally accepted standard-of-care exists; multimodality regimens combining surgery, radiotherapy, and chemotherapy are commonly used but remain suboptimal. Targeted strategies leveraging the epigenetic dependencies of NUT carcinoma (e.g., BET inhibition) are under active investigation, yet robust and generalizable clinical evidence is still evolving (8–10).

At the molecular level, NUT carcinoma is defined by *NUTM1* fusions (11), but the spectrum of fusion partners is increasingly diverse. Beyond canonical partners such as BRD4, BRD3, and NSD3, multiple zinc-finger proteins have been reported, and emerging data continue to expand the list to include non-BET partners such as MGA, YAP1, and CIC (3). This expanding fusion landscape raises the possibility of fusion partner–specific predilections for histologic lineage and anatomical site, which may contribute to phenotypic heterogeneity and further complicate real-world recognition, classification, and risk stratification. However, evidence addressing these relationships remains fragmented across case reports, small clinicopathologic series, and mechanistic studies. Consequently, there is a persistent lack of (i) a systematic, quantitative synthesis of fusion partner–histology–anatomy associations and (ii) an integrative framework that connects research trends, mechanistic insights, and clinical diagnostic challenges, particularly for the thoracic and head and neck presentations that account for a substantial proportion of reported cases.

Bibliometric analysis provides a quantitative and reproducible approach to mapping knowledge structures, collaboration networks, and the temporal evolution of research hotspots within a scientific field (12, 13). Nevertheless, bibliometric evidence specifically characterizing the intellectual landscape and thematic evolution of thoracic and head and neck NUT carcinoma remains limited. Moreover, how bibliometrically identified “frontier themes” align with—and potentially inform—major clinical unmet needs such as misdiagnosis, diagnostic delay, and fusion-driven heterogeneity has not been systematically articulated. Therefore, we aimed to: (i) delineate the knowledge structure, collaboration networks, and temporal evolution of research hotspots in WoSCC-indexed thoracic and head and neck NUT carcinoma literature using bibliometric approaches; (ii) synthesize WoSCC-indexed clinicopathologic evidence to quantify anatomical distribution, high-frequency histologic features, and patterns of initial misdiagnosis; (iii) explore associations between *NUTM1* fusion partners and tissue-origin/histologic lineage as well as anatomical site; (iv) interpret these associations in the context of current mechanistic models and emerging multimodal technologies; and (v) assess whether major literature-derived clinicopathologic and immune-biomarker signals, particularly *BRD4::NUTM1* predominance and PD-L1 negativity, could be corroborated in an independent institutional cohort.

## 2 Methods

### 2.1 Data source, search strategy, and corpus definition

**Figure 1:**
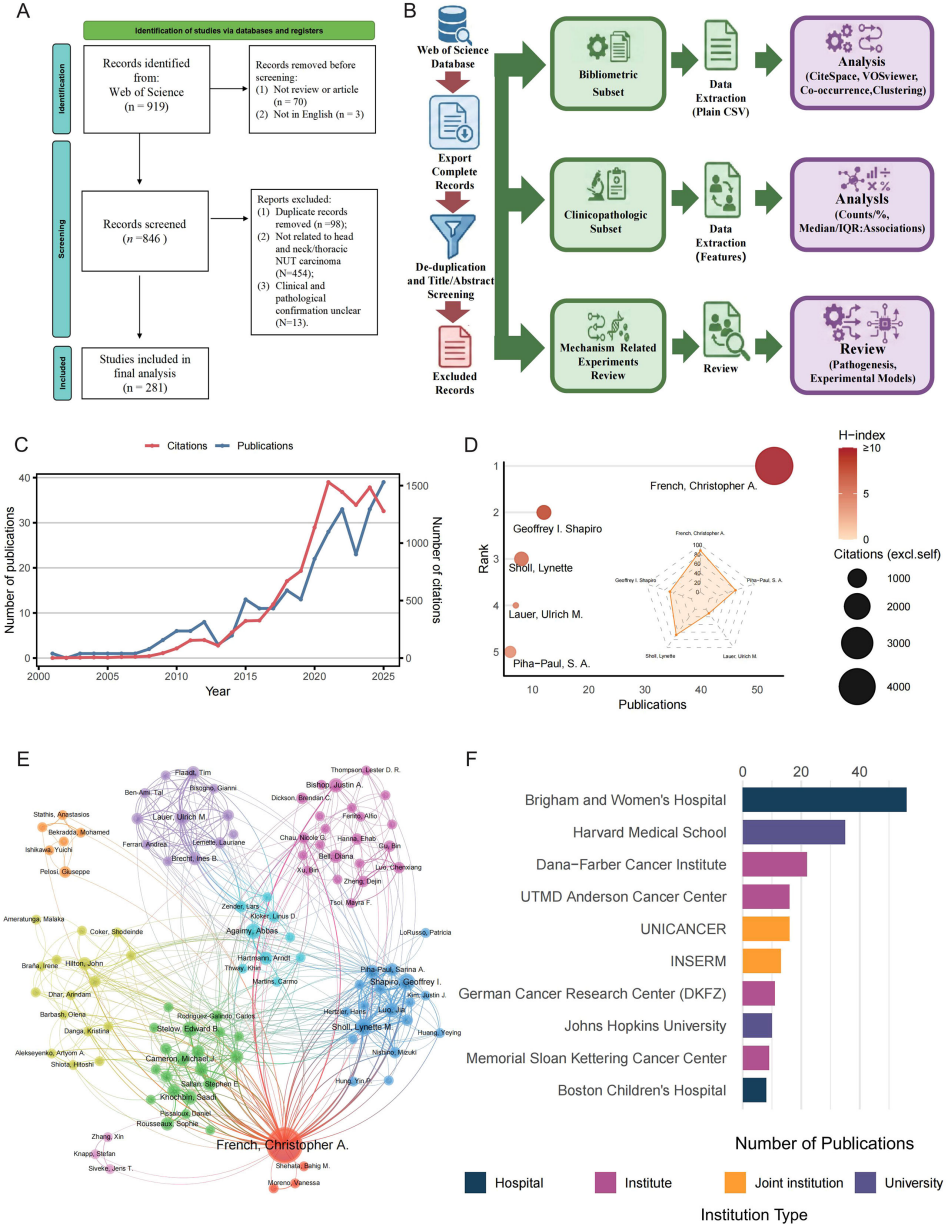
Study design and publication dynamics of NUT carcinoma literature (WoSCC, 1991–2025). **(a)** PRISMA 2020 flow diagram showing record identification, screening, eligibility assessment, and included analytic subsets. **(b)** Study workflow and prespecified subsets: bibliometrics; clinicopathologic synthesis (case-level fusion/site/phenotype extraction); and mechanistic/technology evidence mapping. **(c)** Annual publications and cumulative growth (1991–2025); quadratic fit of cumulative publications (R² = 0.9868) indicates accelerated growth after 2015. **(d)** Top productive authors (by publication count), led by FRENCH CA. **(e)** Co-authorship network highlighting major collaboration hubs. **(f)** Institutional contribution map showing concentration of high-output/high-impact centers (including Harvard-affiliated institutions).

We used the Science Citation Index Expanded (SCI-EXPANDED) within the Web of Science Core Collection (WoSCC) as the sole source database and applied a unified search strategy (fields and terms in **Supplementary Table 1**) to retrieve English-language publications from January 1, 1991 to December 31, 2025(executed on January 4, 2026), yielding 919 records. WoSCC was selected *a priori* because the bibliometric component required standardized citation metadata, complete records, and cited-reference information compatible with CiteSpace, VOSviewer, and bibliometrix. This single-source strategy also ensured that the bibliometric mapping and the downstream case-level synthesis were derived from a unified and reproducible corpus. PubMed/MEDLINE, Embase, and Scopus were not additionally searched because combining multiple citation databases would require extensive cross-database harmonization, deduplication of records, and reconciliation of non-equivalent cited-reference metadata, which could introduce database-dependent inconsistencies into co-citation, collaboration, and thematic evolution analyses. We acknowledge, however, that this strategy may reduce case capture for the clinicopathologic synthesis and may affect the representativeness of pooled estimates, particularly for rare case reports not indexed in WoSCC. Briefly, the WoSCC topic search combined three concept blocks: (1) NUT carcinoma/*NUTM1*-related terms, including NMC, “NUT carcinoma,” “NUT midline carcinoma,” “*NUTM1* fusion,” “*NUTM1*-rearranged,” “*NUTM1*-rearranged carcinoma,” “NUT rearrangement,” “Nuclear protein in testis,” “*NUTM1* protein,” and recurrently reported fusion partners such as BRD4, BRD3, NSD3, ZNF532, and ZNF592; (2) malignancy-related terms, including cancer, carcinoma, and neoplasm; and (3) thoracic and head and neck anatomical terms, including lung, pulmonary, bronchial, tracheal, pleural, mediastinal, thymic, esophageal, chest-wall, “head and neck,” “head & neck,” “upper aerodigestive tract,” HNSCC, sinonasal, nasal/paranasal sinus, pharyngeal, laryngeal, oral cavity, salivary gland, cervical/neck, thyroid/parathyroid, external auditory canal, temporal bone, and skull-base terms. The full line-by-line WoSCC search strategy, including Boolean combinations and search counts, is provided in **Supplementary Table 1**. The study selection process is shown in the PRISMA 2020 flow diagram in **Figure 1A**, and the completed PRISMA 2020 checklist is provided as a separate supplementary checklist.

All records were exported in the format of ‘Complete Records and Cited References’ to build the main dataset. Subsequently, publication-level deduplication was performed based on DOI, title, first author, and publication year, followed by title/abstract screening for relevance. For the clinicopathologic synthesis, patient-level deduplication was further conducted to minimize duplicate case inclusion across case reports and series by cross-checking institution, author group, publication year, age, sex, primary site, fusion partner, histology, treatment history, and outcome information. When overlapping reports were suspected, the most complete or most recent report was retained for case-level extraction, while earlier or less complete reports were used only for contextual information when necessary. This process resulted in a final set of 281 publications that met the analysis criteria. To focus on thoracic and head and neck disease, we included publications whose title/abstract/keywords explicitly addressed thoracic or head and neck NUT carcinoma. From the same eligible corpus, we predefined four analytic subsets (**Figure 1B**): (1) a bibliometric subset (original articles and reviews) for mapping knowledge structure, thematic evolution, and collaboration patterns; (2) a clinicopathologic synthesis subset including studies enabling extraction of case-level variables (fusion partner, primary anatomical site, and histologic descriptors for harmonized stratification); (3) an immune-biomarker subset to abstract PD-L1 expression and TMB/MSI status (and immunotherapy exposure/outcome descriptors where available); and (4) a narrative evidence-mapping component in the Discussion focusing on representative literature on pathogenesis, model construction, multimodal diagnostics, and evolving therapeutic modalities (with emphasis on immunotherapy).

We assembled an independent retrospective institutional cohort of thoracic and head and neck NUT carcinoma cases to provide clinical validation beyond published reports. This cohort was analyzed separately from the bibliometric and literature-synthesis datasets and was used specifically to assess the reproducibility of the major clinicopathologic and immune-biomarker signals identified in the pooled literature, with emphasis on fusion-partner distribution, PD-L1 landscape, and clinical aggressiveness.

### 2.2 Data extraction

For data extraction, bibliometric records were converted into standardized formats (plain text and CSV), and key fields were extracted, including title, authorship, affiliations, country/region, journal, publication year, keywords (author keywords and database-generated keywords), cited references, and citation metrics for descriptive statistics and network analyses. For clinicopathologic synthesis, two experienced pathologists independently abstracted case-level variables using a prespecified extraction form, with cross-checking and adjudication. Variables included fusion status (fusion partner and/or confirmatory evidence of *NUTM1* rearrangement), primary anatomical site, histopathological descriptors enabling classification, diagnostic pathway variables (initial diagnosis category), and major treatment/outcome descriptors when available. To improve comparability across heterogeneous reports, cases were further mapped into harmonized anatomical regions and histologic groupings (squamous vs non-squamous), with disagreements resolved by consensus and third-pathologist adjudication when necessary. For immune-biomarker abstraction, we extracted PD-L1 expression results, including assay clone, scoring system, and cut-off when reported. PD-L1 results were harmonized into three descriptive categories: Negative, Low expression, and High expression. Author-reported categorical interpretations were prioritized. When only numeric results were available, cases were categorized as Negative if TPS was <1% or CPS was <1, Low expression if TPS was ≥1% to <30% or CPS was ≥1 to <30, and High expression if TPS was ≥30% or CPS was ≥30. Because TPS is reported as a percentage of viable tumor cells with membranous staining, whereas CPS is a unitless score incorporating staining in tumor cells and selected immune cells, these categories were used primarily for descriptive comparability rather than as directly interchangeable quantitative thresholds. Given the heterogeneity of PD-L1 assays, scoring systems, and reporting thresholds, we additionally used a binary sensitivity framework classifying cases as PD-L1 Negative versus Any expression. Author-reported negative cases, TPS <1%, or CPS <1 were classified as Negative, whereas author-reported low/high expression, TPS ≥1%, or CPS ≥1 were classified as Any expression. When both TPS and CPS were available, TPS was prioritized for tumor-cell expression, whereas CPS was retained descriptively and used only in the binary sensitivity framework.

We additionally extracted MSI status (categorized as MSS vs MSI-H/MSI-L when reported) and tumor mutational burden (TMB, mut/Mb) as a continuous variable. Due to heterogeneous reporting and substantial missingness across case reports, immune-biomarker analyses were performed using endpoint-specific complete-case datasets.

### 2.3 Bibliometric and clinicopathologic analyses, quality assessment

For analysis, bibliometric mapping and visualization were performed using CiteSpace,VOSviewer and R-bibliometrix, including keyword co-occurrence, clustering, and burst detection to identify thematic structure, evolving hotspots, and emerging fronts, as well as collaboration networks across authors, institutions, and countries/regions. In the clinicopathologic synthesis, categorical variables are reported as counts (%), and continuous variables as median (IQR) and/or mean (95% CI), as appropriate. Associations between fusion partners and clinicopathologic strata, including histologic grouping, anatomical region, anatomical-pathologic strata, and immune-biomarker categories, were assessed using Fisher’s exact test and reported as odds ratios (ORs) with 95% confidence intervals (CIs). Missing data were handled using endpoint-specific complete-case analyses. For PD-L1, both the prespecified three-category harmonized framework and a binary negative-versus-any-expression framework were summarized. Because analyses across fusion partners, anatomical strata, histologic categories, and immune-biomarker groups were exploratory and hypothesis-generating in the setting of a very rare disease, we did not apply formal multiplicity correction. All reported p values are two-sided and unadjusted; statistically significant associations, particularly those involving rare fusions, should therefore be interpreted as exploratory enrichment signals rather than confirmatory evidence. Methodological quality was appraised using the Agency for Healthcare Research and Quality (AHRQ) approach where applicable (**Supplementary Table 2**) (14). Because the available evidence consisted predominantly of case reports and small case series, the AHRQ assessment was used descriptively to characterize reporting completeness and inherent risk of bias rather than as an exclusion criterion. No predefined AHRQ score threshold was applied for study exclusion. Studies were retained if they met the eligibility criteria and provided extractable case-level clinicopathologic, fusion, or immune-biomarker data.

### 2.4 Independent clinical validation cohort

We retrospectively enrolled pathologically confirmed NUT carcinoma cases from our institution for independent cohort validation. Eligible cases were required to have at least one of the following available: fusion-partner data, PD-L1 testing data, or immunotherapy exposure history. Extracted variables included age, sex, smoking history, primary site, clinical stage, histologic category, fusion partner, PD-L1 status, Ki-67 proliferation index, and immunotherapy exposure history. Fusion-partner annotation was determined, when available, based on NGS RNA fusion panel results. PD-L1 expression in the institutional cohort was assessed uniformly using the 22C3 pharmDx assay and tumor proportion score (TPS). For consistency with the literature-derived three-tier harmonization, institutional PD-L1 results were descriptively mapped as Negative (<1%), Low expression (1% to <30%), and High expression (≥30%). Raw TPS values were retained for interpretation, and the binary framework, Negative versus Any expression, was additionally applied as a sensitivity analysis. Exact binomial tests were used to compare observed proportions in the institutional cohort with the corresponding WoSCC-indexed literature-derived estimates, and Fisher’s exact test was used for subgroup comparisons. All statistical analyses were performed using R version 4.3.x, with two-sided *p* < 0.05 considered statistically significant.

## 3 Results

### 3.1 Bibliometric analysis of NUT carcinoma research

#### 3.1.1 Dataset characteristics and annual publication trends

This study retrieved 281 thoracic and head and neck NUT carcinoma-related publications from the Web of Science Core Collection (SCI-EXPANDED), distributed across 143 journals. The document types were predominantly original research articles (224 articles) and review articles (57 reviews). The dataset comprised 1,863 authors with 2,264 author appearances, averaging 8.06 authors per publication. Single-authored publications accounted for only 6 papers (2.14%), while internationally collaborative papers represented 19.93%, reflecting the highly multidisciplinary collaborative nature of this field. The publications contained 3,539 references, 533 author keywords (DE), and 384 KeyWords Plus (ID). The total citation count reached 11,589 times, with an average of 41.24 citations per publication, an annual citation intensity of 3.981 citations per publication per year, and a median citation count of 10. Zero-citation publications accounted for 16.37% (n=46), indicating a typical long-tail distribution where highly cited core publications coexist with a substantial proportion of low- or zero-citation research.

Annual publication volume analysis revealed a sustained growth trend in NUT carcinoma research, with an average annual growth rate of 16.49%. During the early phase (1991–2014), publication output was low and fluctuating; publications were sporadic before 2001, followed by gradual accumulation with annual outputs typically ranging from 1 to 8 papers. Research entered a growth phase in 2015 (13 papers) and accelerated markedly after 2020, remaining high during 2022–2025. A quadratic polynomial fitting model based on cumulative publication volume (y = 0.75X² − 9.44X + 28.25) demonstrated excellent goodness-of-fit (R² = 0.9868), supporting a sustained upward trajectory in research output (**Figure 1C**).

#### 3.1.2 Core authors, institutions, and national contribution patterns

**Figure 2:**
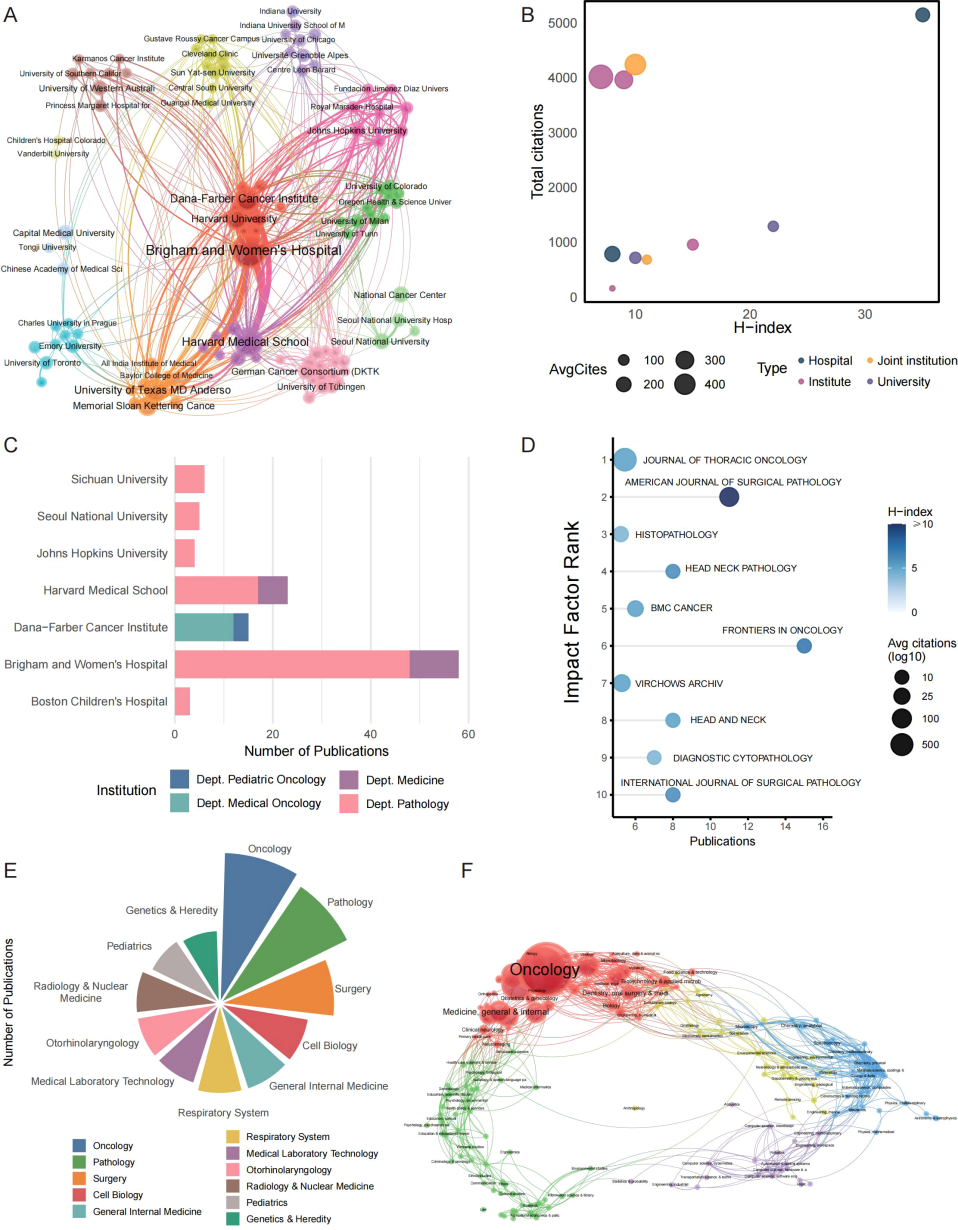
Institutional and journal landscape of thoracic and head and neck NUT carcinoma research. **(a)** Inter-institutional collaboration network showing major collaborative clusters. **(b)** Impact of the top 10 institutions (total citations vs H-index; bubble size = average citations per paper). **(c)** Department-level contributions within top institutions, dominated by pathology and oncology. **(d)** Most productive journals by publication count. **(e)** Subject-field distribution of publishing journals. **(f)** Dual-map overlay of subject categories illustrating cross-disciplinary citation links between molecular/biological sciences and clinical medicine.

Author contribution analysis identified FRENCH CA as the most prolific researcher with 53 publications, the highest average citations per paper, and the most extensive collaborative network (**Figures 1D, E**), followed by SHAPIRO GI (12 papers), SHOLL LM (8 papers), and LAUER UM (7 papers).Research was concentrated in top-tier U.S. medical centers (**Figure 1F**), with Harvard-affiliated institutions (Harvard Medical School, Brigham & Women’s Hospital, Massachusetts General Hospital, Boston Children’s Hospital) leading globally through 60 publications (21.35%), 8,428 citations (140.47 per paper vs. field average 41.24), and an H-index of 35. The University of Texas MD Anderson Cancer Center was another major contributor. Co-organization networks revealed dense collaborations centered around Harvard institutions (**Figure 2A**). **Figure 2B** further visualizes the comparative impact of the top-10 institutions by plotting total citations versus H-index (bubble size = average citations per paper), underscoring the concentration of high-impact output in leading U.S. centers. Department-stratified analysis shows that publications from the top-10 institutions are primarily contributed by pathology and oncology departments (**Figure 2C**), and department-level impact—summarized by total citations vs H-index with bubble size denoting average citations—clusters in a subset of high-performing units (**Supplementary Figure 1**).

**Figure 3:**
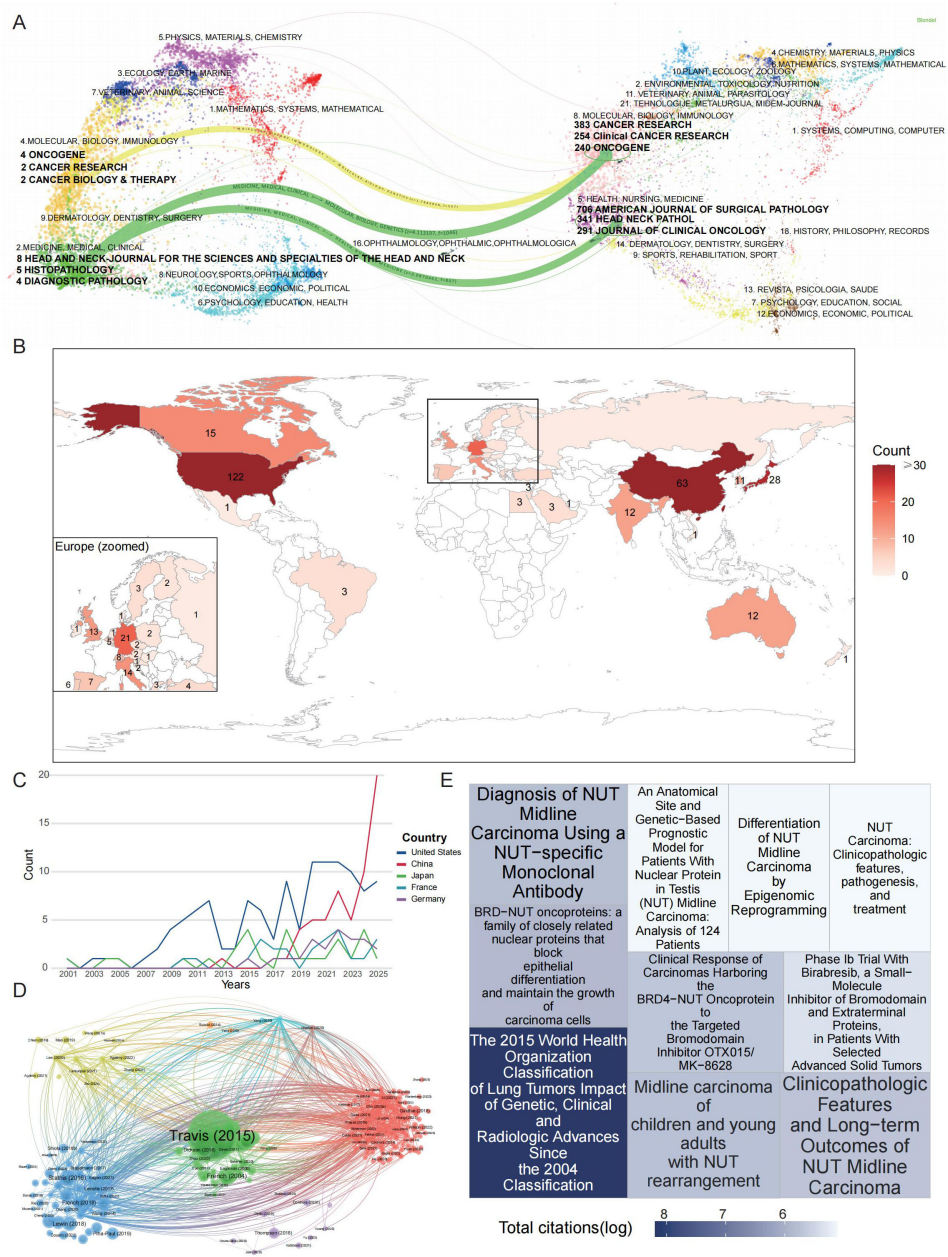
Citation structure and geographic contributions in NUT carcinoma research. **(a)** Dual-map overlay indicating citation flow from molecular/biological sciences to clinical medicine. **(b)** Country/region contributions by output and citation impact, led by the United States.(c) Temporal publication trends of major contributing countries/regions, with recent rapid growth in China. **(d)** Co-citation/coupling network identifying the core knowledge base in diagnostics, fusion biology, and epigenetic mechanisms. **(e)** Top-cited landmark publications supporting the field’s diagnostic and mechanistic framework.

Regarding journal distribution, major publishing journals (**Figure 2D**) were concentrated in the fields of oncology and pathology (**Figure 2E**), and the subject-category overlay further indicates that NUT carcinoma research is positioned at the interface between clinical oncology/pathology and molecular biology disciplines (**Figure 2F**). Frontiers in Oncology (15 papers) and American Journal of Surgical Pathology (11 papers) being the primary sources, followed by Head & Neck Pathology, head and neck, and International Journal of Surgical Pathology (8 papers each). Bradford’s Law (15) analysis identified 14 core zone journals, constituting the primary vehicles for disseminating literature on this topic and demonstrating a pronounced journal clustering effect. The dual-graph overlay analysis of the journal shows that cited literature mainly comes from two major disciplinary clusters: MEDICINE, MEDICAL, CLINICAL and MOLECULAR, BIOLOGY, IMMUNOLOGY. The flow of knowledge presents a unidirectional pattern from the right MOLECULAR, BIOLOGY, IMMUNOLOGY cluster to the left MEDICINE, MEDICAL, CLINICAL cluster, reflecting the knowledge transfer pathway from basic research to clinical translation (**Figure 3A**).

National contribution analysis showed the United States dominated with 99 publications(35%), 8,678 total citations, and 87.66 citations per publication, leading in both output and impact (**Figure 3B**). China ranked second with 59 papers (accounting for 21%), but each paper was cited only 7.80 times on average, indicating that while its output is substantial, its influence is limited. However, in recent years, the growth rate of its literature has been significantly higher than that of other countries(**Figure 3C**).Other major contributors included Japan (22), Germany (17), and France (14).

International collaboration patterns revealed significant disparities: France (MCP ratio 0.50) and Germany (0.3529) demonstrated high transnational collaboration, while China showed minimal international engagement (MCP 3.39%, SCP 96.61%). Notably, France achieved 45.64 citations per publication despite moderate output, reflecting its significant contribution to foundational knowledge.

#### 3.1.3 Citation patterns and knowledge foundation

High-citation literature analysis and bibliographic coupling identified milestone studies and core knowledge frameworks (**Figure 3D**). The temporal heatmap of the top 10 most-cited papers (**Supplementary Figure 2**) further illustrates sustained citation accumulation after 2015, with several seminal works showing pronounced citation bursts during 2018–2025, consistent with the consolidation of a core knowledge base in the field. By total citations (**Figure 3E**), among the cited references in our corpus, the most globally cited work was TRAVIS WD et al.’s 2015 lung cancer classification guidelines (Journal of Thoracic Oncology, 3,613 citations) (2), followed by FRENCH CA et al.’s 2008 Oncogene paper (349) (16), HAACK H et al.’s 2009 American Journal of Surgical Pathology paper (325) (6), and FRENCH CA et al.’s 2004 Journal of Clinical Oncology paper (323) (17). Co-citation analysis identified core publications repeatedly cited within the dataset (**Supplementary Figure 3**): BAUER DE 2012 (Clinical Cancer Research, Freq=164) (18), HAACK H 2009 (Freq=160) (6), FRENCH CA 2004 (Freq=142) (17), and FRENCH CA 2008 (Freq=111) (16), constituting the field’s core knowledge framework in establishing NUT carcinoma molecular characteristics, identifying chromosomal translocations/fusion genes, establishing diagnostic paradigms, and exploring therapeutic approaches. These highly cited publications primarily focused on the oncogenic mechanisms of *BRD4::NUTM1* fusion proteins, epigenetic alterations involving histone hyperacetylation, establishment of diagnostic criteria, and exploration of targeted therapeutic strategies such as BET inhibitors, laying a solid theoretical foundation for subsequent research.

#### 3.1.4 Research hotspots, thematic clustering, and temporal evolution

**Figure 4:**
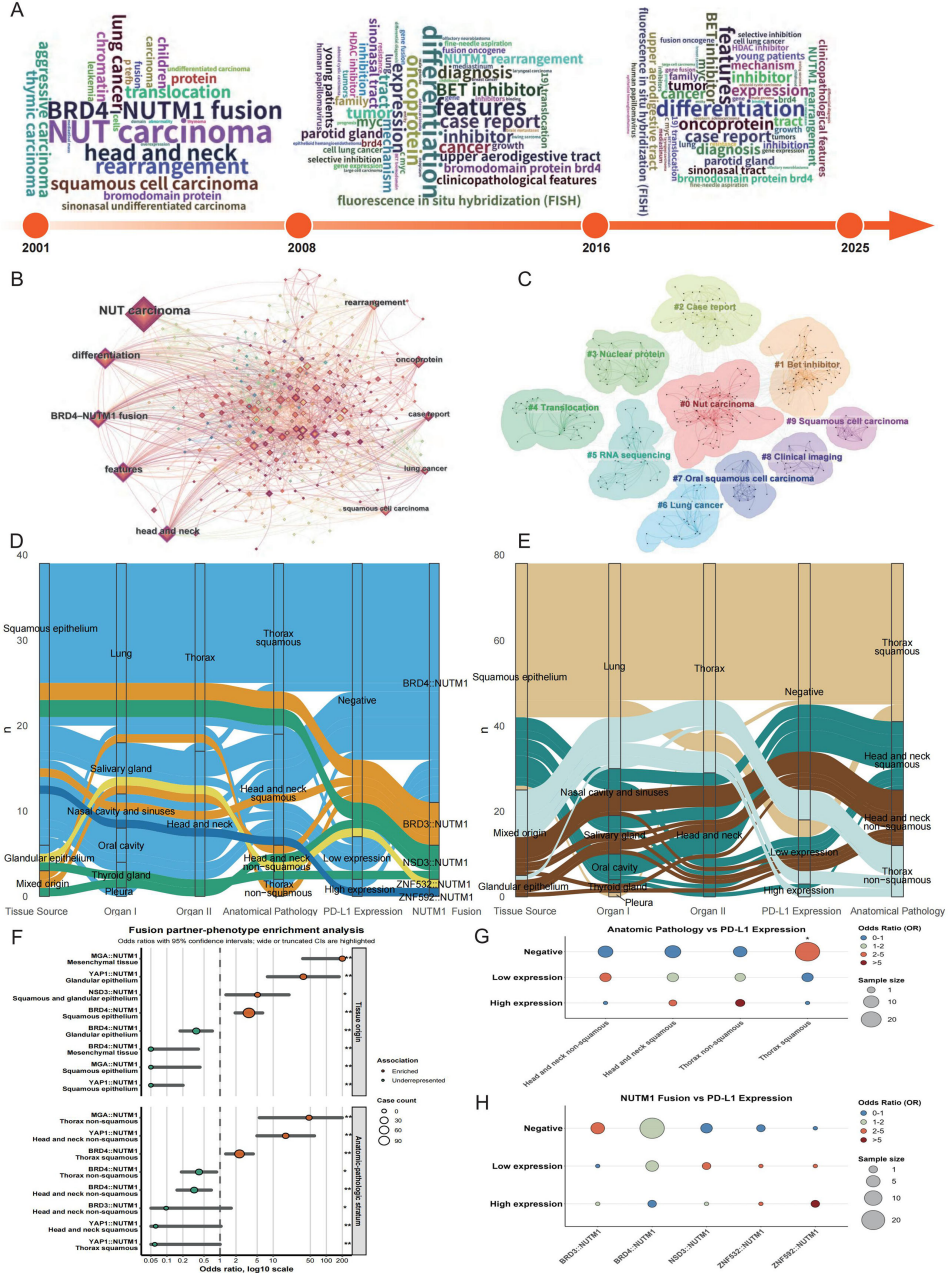
Hotspots and fusion partner–phenotype enrichment in thoracic and head and neck NUT carcinoma. **(a)** Time-sliced keyword word clouds across the study period; word size reflects keyword frequency. **(b)** Keyword co-occurrence network with fusion-related terms as central hubs. **(c)** Keyword clustering map summarizing major thematic domains. **(d)** Clinicopathologic synthesis overview (n=229 cases): distribution of fusion types across anatomic and lineage strata. **(e)** PD-L1 expression across anatomic–lineage strata in thoracic and head-and-neck NUT carcinoma. **(f)** Forest plot showing exploratory enrichment of selected NUTM1 fusion partners across tissue-origin and anatomic–pathologic strata. Odds ratios with 95% confidence intervals are displayed on a logarithmic scale. Point size reflects the number of cases within each stratum. Wide confidence intervals, particularly for rare fusion partners, reflect sparse denominators and statistical imprecision; therefore, associations involving rare fusion partners should be interpreted as exploratory enrichment signals rather than definitive effect-size estimates. Asterisks denote statistically significant associations (**p < 0.01, *p < 0.05). **(g)** Anatomic–pathologic strata versus PD-L1 expression. **(h)** NUTM1 fusion partner versus PD-L1 expression.

Time-sliced keyword word clouds revealed highly concentrated research themes and a clear temporal shift in thoracic and head and neck NUT carcinoma research (**Figure 4A**). The keyword co-occurrence network highlights ‘NUT carcinoma’ and fusion-related terms (e.g., *BRD4::NUTM1*, rearrangement) as central hubs with dense connectivity, reflecting the fusion-centric knowledge structure of the field (**Figure 4B**). Author keywords (DE) centered on “NUT carcinoma” (95 occurrences) and “NUT midline carcinoma” (71 occurrences), with high-frequency accompanying terms including “case report” (26 occurrences), “*NUTM1*” (23 occurrences), “BRD4-NUT” (17 occurrences), “immunohistochemistry” (15 occurrences), “immunotherapy” (11 occurrences), and “next-generation sequencing” (8 occurrences). Among KeyWords Plus (ID), “midline carcinoma” (107 occurrences), “differentiation” (73 occurrences), “outcomes” (58 occurrences), “rearrangement” (43 occurrences), and “BRD-NUT” (33 occurrences) occupied dominant positions, indicating that the research framework primarily revolves around disease entity, differentiation blockade/epigenetic aberrations, chromosomal rearrangements, and clinical outcomes.

Citation burst analysis further revealed temporal shifts in research hotspots (**Supplementary Table 3**). Early bursts concentrated on disease entity and translocation identification-related terms, including “thymic carcinoma” (2003–2013, Strength=4.82), “aggressive carcinoma” (2004–2013, Strength=6.83), and “translocation” (2004–2013, Strength=5.49). Mid-phase bursts featured fusion event and detection-related terminology, with “rearrangement” (2008–2017, Strength=7.61) and “*BRD4::NUTM1* fusion” (2008–2012, Strength=4.29) indicating deepened fusion gene detection and molecular characterization. Recent bursts shifted toward precision diagnostics and targeted strategies, with “bromodomain inhibitor” (2020–2023, Strength=2.41) and “next-generation sequencing” (2021–2022, Strength=2.32) reflecting advances in therapeutic exploration and molecular diagnostic technologies. Notably, “NUT carcinoma” exhibited the strongest burst during 2022–2025 (Strength=11.96), suggesting further reinforcement of disease nomenclature standardization and research attention in recent years.

Thematic clustering analysis identified 11 major research directions spanning entity definition/translocation and fusion discovery, molecular diagnostics, site-stratified pathology, and therapeutic themes including BET/HDAC inhibition (**Figure 4C**: Keyword clustering map; details in **Supplementary Table 4**). Temporal evolution analysis revealed a four-phase development pattern. The Foundational Phase (pre-2010) was dominated by NUT carcinoma and Translocation themes, with a pivotal node around 2005 marking disease entity establishment and identification of t(15;19) translocation/BRD4-NUT fusion gene, laying the groundwork for subsequent molecular characterization. The Expansion Phase (2010-2015) witnessed thematic diversification, with the prominent emergence of BET inhibitor during 2012–2015 signaling a transition from mechanistic exploration to therapeutic strategies, while sustained growth in Case report, Nuclear protein, and RNA sequencing reflected parallel advancement in case accumulation, mechanistic research, and diagnostic technologies. The Diversification Phase (2015-2020) demonstrated multi-directional expansion, with the nodal appearance of RNA sequencing around 2015 indicating widespread adoption of next-generation sequencing, the emergence of Lung cancer and Oral squamous cell carcinoma representing deepened site-specific investigations, and Clinical imaging (2018-2020) reflecting enhanced emphasis on radiological features. The Contemporary Phase (2020-2025) emphasized precision medicine translation, with NUT carcinoma, BET inhibitor, Case report, and RNA sequencing maintaining continuous activity and increasingly strengthened cross-cluster connections, forming an integrated research paradigm encompassing molecular diagnostics, targeted therapies, and clinical management. The field evolved from disease definition and molecular characterization (2001-2010) through therapeutic exploration (2010-2015) to precision medicine implementation (2015-2025), with case documentation and molecular diagnostics as persistent themes. This trajectory is corroborated by keyword timeline analysis showing both emerging clusters and enduring core themes across 2001-2025 (**Supplementary Figure 4**).

#### 3.1.5 Clinical problem-oriented thematic distribution

To enhance clinical interpretability of results, automatic thematic annotation statistics were performed on titles-abstracts-keywords (categories allowed to overlap). Fusion gene-related research comprised 173 papers (61.57%), with *BRD4::NUTM1*-related studies dominating at 82 papers (29.18%), followed by t(15;19) chromosomal translocation at 53 papers (18.86%), and *BRD3::NUTM1* and *NSD3::NUTM1* each at 12 papers (4.27%), reflecting the central role of fusion gene detection in diagnosis and molecular subtyping. Regarding treatment strategies, chemotherapy (99 papers, 35.23%) and radiotherapy (82 papers, 29.18%) showed the highest frequencies, indicating that conventional treatments remain the clinical mainstream; immunotherapy (63 papers, 22.42%) demonstrated rapid growth; BET inhibitors (31 papers, 11.03%) and HDAC inhibitors (9 papers, 3.20%) represented frontier explorations in targeted and epigenetic therapies. Research types were predominantly case reports/case series (93 papers, 33.10%), reflecting disease rarity and the importance of clinical experience accumulation; cohort studies (32 papers, 11.39%) and basic research (33 papers, 11.74%) were comparable; clinical trials (21 papers, 7.47%) were limited in number, indicating scarcity of high-quality prospective studies.

### 3.2 Systematic clinicopathologic synthesis and fusion partner–phenotype associations

Guided by Section 3.1 showing persistent emphasis on rearrangements/fusions and molecular diagnostics, we conducted a systematic clinicopathologic synthesis using the prespecified clinicopathologic subset from the same WoSCC corpus (Methods 2.1, Subset 2). Overall, 109 studies were included, yielding 229 thoracic and head and neck NUT carcinoma cases with extractable core clinicopathologic annotations and definitive fusion types. The anatomical distribution was nearly balanced between the head and neck and thorax (50.22%, 115/229 vs. 49.78%, 114/229): thoracic cases most commonly involved the lung (34.06%, 78/229) and mediastinum (10.92%, 25/229), whereas head and neck cases were enriched in the salivary glands (13.97%, 32/229), nasal cavity/sinuses (13.10%, 30/229), and thyroid gland (8.73%, 20/229). Histogenetic review indicated predominance of squamous epithelial origin (71.62%, 164/229), followed by glandular epithelium (17.03%, 39/229), mesenchymal tissue (4.80%, 11/229), biphenotypic squamous-and-glandular differentiation (3.93%, 9/229), and mixed origin (2.62%, 6/229).The overall mapping from tissue origin and sites to anatomical–pathologic strata and fusion types is summarized in **Figure 4D**.

The fusion landscape was dominated by *BRD4::NUTM1* (64.19%, 147/229), followed by *NSD3::NUTM1* (14.85%, 34/229) and *BRD3::NUTM1* (10.92%, 25/229), with additional cases mostly harboring *YAP1::NUTM1* (5.68%, 13/229) and *MGA::NUTM1* (3.06%, 7/229).Under the prespecified Fisher’s exact and odds-ratio framework (Methods 2.3), fusion partners exhibited non-random distributions across histogenetic and anatomical–pathologic strata (**Figure 4F**; **Supplementary Figure S5**, **S6**). *BRD4::NUTM1* was significantly associated with squamous epithelial origin (OR = 3.49,*p=*0.0001) and enriched in the thoracic squamous subgroup (OR = 2.33,*p=*0.0063), while being underrepresented in thoracic non-squamous and head and neck non-squamous subgroups (OR = 0.40, *p=*0.0256; OR = 0.33, *p=*0.0034). In contrast, *YAP1::NUTM1* was strongly associated with glandular epithelial origin (OR = 36.93,*p<*0.001) and markedly enriched in the head and neck non-squamous subgroup (*p<*0.001), with no cases observed in squamous-related strata; *MGA::NUTM1* showed an extremely strong association with mesenchymal origin (*p<*0.001) and was concentrated in the thoracic non-squamous subgroup (*p<*0.001). Collectively, these results suggest fusion partner–associated enrichment patterns across lineage and anatomical–pathologic stratification. However, because several non-BRD4 fusion groups were represented by small case numbers and wide confidence intervals, these findings should be interpreted as exploratory and hypothesis-generating rather than deterministic or mechanistically validated associations. In addition, recurrent microscopic patterns included sheet-like and nested architectures in both thoracic and head and neck tumors, with prominent nucleoli frequently reported (**Supplementary Table 5**). Abrupt keratinization was documented more often in head and neck tumors than in thoracic tumors (**Supplementary Table 5**). Finally, the most common initial diagnoses were squamous cell carcinoma (36.7%), undifferentiated tumor (16.2%), and poorly differentiated carcinoma (14.8%) (**Supplementary Table 6**).

### 3.3 Immune-biomarker landscape

Motivated by the bibliometric signal of increasing emphasis on immunotherapy and tumor microenvironment–related themes (Section 3.1), we further synthesized immunotherapy-relevant biomarkers from the clinicopathologic corpus.MSI status and numeric TMB values were available in 16 cases. All 16 were MSS. TMB values were generally low (median 1.35 mut/Mb; range 0–7.6 mut/Mb), with no cases reaching commonly used “high-TMB” thresholds.

PD-L1 status was extractable in 78 thoracic/head and neck cases. Overall, PD-L1 was Negative in 60/78 (76.92%), Low expression in 13/78 (16.67%), and High expression in 5/78 (6.41%). Across anatomical–pathologic strata, PD-L1 negativity was particularly prevalent in the thorax squamous subgroup (33/37, 89.19%), which showed increased odds of PD-L1 negativity compared with other strata (OR = 4.28, 95%CI 1.26–14.52; *p=*0.017) (**Figure 4E**; **Figure 4G**). Tissue-origin groupings and broad anatomical region (thorax vs head and neck) were not significantly associated with PD-L1 category in this dataset. To test whether the predominance of PD-L1 negativity was robust to simplified PD-L1 harmonization, we performed a binary sensitivity analysis. In the literature-derived PD-L1 subset, 60 of 78 cases were classified as PD-L1 Negative and 18 of 78 as Any expression, confirming the predominance of PD-L1 negativity. Across anatomical–pathologic strata, thoracic squamous tumors included 33 PD-L1-negative and 4 PD-L1-expressing cases, whereas the remaining strata included 27 PD-L1-negative and 14 PD-L1-expressing cases. These binary sensitivity results were consistent with the three-category analysis and are summarized in **Supplementary Table 7**.

Among 39 cases with both definitive fusion-partner annotation and PD-L1 status, PD-L1 was predominantly Negative (32/39, 82.1%), with Low expression in 5/39 (12.8%) and High expression in 2/39 (5.1%) (**Figure 4H**). No statistically significant association between the descriptive three-tier PD-L1 category and fusion partner was detected, although interpretability was limited by sparse counts for non-BRD4 and rare fusion groups. To address the heterogeneity of PD-L1 reporting across studies, we further performed a binary sensitivity analysis in which PD-L1 status was classified as Negative versus Any expression. Among the 39 literature-derived cases with both definitive fusion-partner annotation and PD-L1 data, 32 cases were PD-L1 negative and 7 showed any PD-L1 expression. By fusion partner, *BRD4::NUTM1* accounted for 28 cases, including 23 PD-L1-negative and 5 PD-L1-expressing tumors; *BRD3::NUTM1* accounted for 5 cases, all of which were PD-L1 negative; and *NSD3::NUTM1* accounted for 4 cases, including 3 PD-L1-negative and 1 PD-L1-expressing tumor. The remaining *ZNF532::NUTM1* and *ZNF592::NUTM1* cases were represented by one case each. Exploratory Fisher’s exact testing did not identify a significant association between fusion partner and binary PD-L1 status (**Supplementary Table 8**). These findings support the predominance of PD-L1 negativity across fusion-defined subsets, while emphasizing that inference for non-BRD4 and rare fusion partners remains limited by sparse case numbers.

### 3.4 Independent cohort validation

We analyzed an independent institutional cohort of 59 pathologically confirmed NUT carcinoma cases. The baseline clinicopathologic characteristics of this cohort are summarized in **Table 1**. The cohort comprised 35 males and 24 females, with a median age of 34 years (IQR, 23.5–46.5; range, 7–76 years). Most patients presented with advanced-stage disease, including 44/59 (74.6%) with stage IV disease and 15/59 (25.4%) with stage III disease. Primary tumor sites were mainly thoracic (39/59, 66.1%) and head and neck (20/59, 33.9%). Eleven patients (18.6%) had a smoking history, and 20 (33.9%) had previously received immunotherapy.

**Table 1:** Baseline clinicopathologic characteristics of the independent institutional cohort.

Characteristic	Overall (N=59)
Age, median (IQR), years	34 (23.5–46.5)
Age range, years	7–76
Sex, male	35 (59.3)
Sex, female	24 (40.7)
Smoking history, yes	11 (18.6)
Tumor size, median (IQR), cm	5.33 (3.55–6.05)
Tumor size range, cm	1.61–22.9
Stage III	15 (25.4)
Stage IV	44 (74.6)
Primary site, thoracic	39 (66.1)
Primary site, head and neck	20 (33.9)
Prior immunotherapy exposure, yes	20 (33.9)
Histologic classification available	39 (66.1)
Squamous cell carcinoma	38 (64.4)
Adenocarcinoma	1 (1.7)
Fusion annotation available	34 (57.6)
*BRD4::NUTM1*	25 (73.5)*
*BRD3::NUTM1*	8 (23.5)*
*NSD3::NUTM1*	1 (2.9)*
PD-L1 available	33 (55.9)
Negative	25 (75.8)*
Low	5 (15.2)*
High	3 (9.1)*
Ki-67 available	44 (74.6)

Data are presented as n (%) unless otherwise indicated. Percentages without an asterisk are calculated using the full cohort denominator (N=59). *Percentages are calculated among assessable cases for the corresponding variable. Fusion-partner percentages are calculated among 34 fusion-annotated cases, and PD-L1 percentages are calculated among 33 PD-L1-tested cases. PD-L1 in the institutional cohort was assessed using the 22C3 pharmDx assay and recorded as TPS. For descriptive comparison with the literature-derived dataset, TPS values were mapped to the same prespecified study-level harmonized categories: Negative (<1%), Low (1% to <30%), and High (≥30%).

Missingness was explicitly quantified in the institutional cohort. Histologic information was available for 39 of 59 patients and missing in 20 of 59 patients (33.9%). Fusion-partner annotation was available in 34 of 59 patients (57.6%), and PD-L1 testing was available in 33 of 59 patients (55.9%). Among fusion-annotated cases, *BRD4::NUTM1* remained the predominant fusion partner (25/34, 73.5%), followed by *BRD3::NUTM1* (8/34, 23.5%) and *NSD3::NUTM1* (1/34, 2.9%). This proportion was not significantly different from the pooled literature-derived estimate (147/229, 64.2%; exact binomial test, *p* = 0.288).

Among PD-L1-tested cases, 25 of 33 tumors were PD-L1 negative, 5 showed low expression, and 3 showed study-level harmonized high expression under the three-tier framework; no TPS-scored institutional case reached the conventional TPS ≥50% high-expression threshold. This was consistent with the literature-derived estimate of PD-L1 negativity in the overall PD-L1-assessable subset (60/78, 76.9%; exact binomial test, *p* = 0.838; **Supplementary Table 7**) and supported the robustness of the immune-cold pattern under a simplified binary framework. However, because histologic information was missing in one-third of the institutional cohort and rare non-BET fusion partners such as *YAP1::NUTM1* and *MGA::NUTM1* were not represented, the institutional cohort was not sufficiently powered to independently validate fine fusion partner–phenotype enrichment patterns observed in the literature-derived analysis.

## 4 Discussion

### 4.1 Key findings

This study integrates bibliometrics with systematic case-level synthesis to map thoracic and head and neck NUT carcinoma. The field has shifted since 2015 toward molecular diagnostics, epigenetic targeting, and immunotherapy. Clinically, cases are mainly squamous but include non-squamous and mixed phenotypes, with exploratory fusion partner–associated enrichment patterns observed across selected lineage and anatomical strata. Most reported tumors are PD-L1–negative and MSS/low-TMB, underscoring the need for prospective, biomarker-integrated registries.

### 4.2 Diagnostic challenges

Building on these findings, we further dissect the persistent causes of misdiagnosis and delayed diagnosis from three perspectives: morphologic clues, lineage/phenotypic spectrum, and testing workflows. Despite growing awareness, misdiagnosis and diagnostic delay remain common (4, 5, 19): (1) Non-specific morphology: NUT carcinoma often presents as poorly differentiated/undifferentiated tumors and substantially overlaps with poorly differentiated squamous cell carcinoma, undifferentiated carcinoma, and some small round cell or sarcomatoid neoplasms; moreover, thoracic and head and neck biopsy specimens are frequently limited by scant tissue, necrosis, and crush artifact, further reducing diagnostic clues. (2) Broad phenotypic spectrum: While focal “abrupt keratinization” can be suggestive, it is not consistently present or may be only focal; in addition, non-squamous or mixed phenotypes broaden the differential diagnosis, making NUT-IHC less likely to be incorporated as a “routine test.” (3) Heterogeneity in testing workflows and thresholds: Institutions vary in the threshold for “when to add NUT-IHC” and “when to escalate to fusion testing for confirmation,” resulting in under-testing and delayed testing.

### 4.3 Mechanistic context

**Figure 5:**
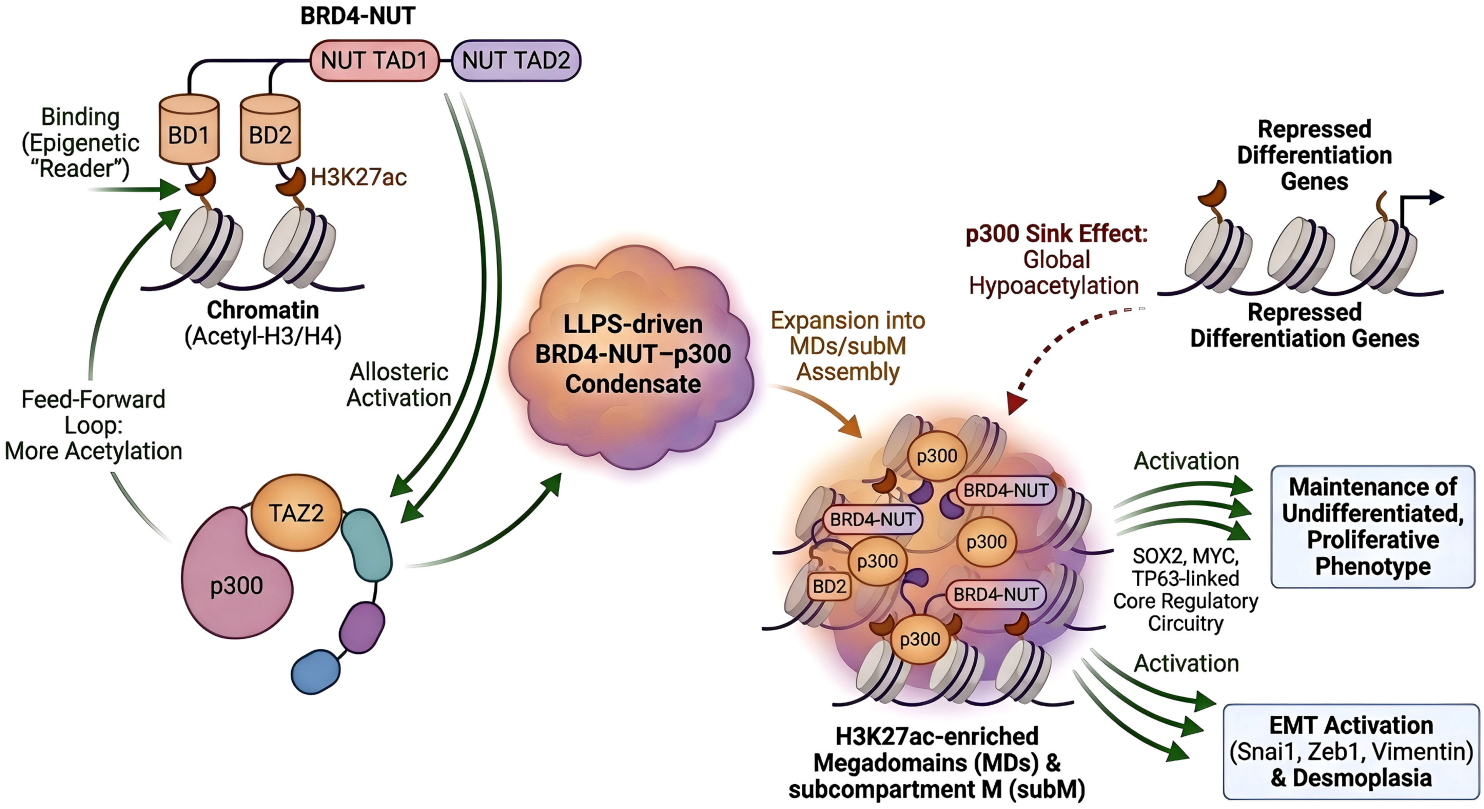
*BRD4::NUTM1-*p300 feed-forward acetylation circuitry and megadomain formation underlying differentiation blockade in NUT carcinoma. LLPS, liquid–liquid phase separation; MDs, megadomains; subM, subcompartment M; EMT, epithelial–mesenchymal transition.

Importantly, delineating the key molecular circuitry driven by fusions not only helps define more actionable triggers for diagnostic testing, but also provides a rationale for subsequent exploration of epigenetic dependency–based therapies. In canonical BRD4/BRD3/NSD3 fusions and a subset of non-canonical fusions, available evidence broadly supports a convergent epigenetic oncogenic logic: *NUTM1* fusion proteins assemble an aberrant “reader–writer platform” that enhances histone acetylation and transcriptional amplification while blocking differentiation programs (11, 20–22), thereby maintaining an undifferentiated, highly proliferative state. As shown in **Figure 5**, bromodomains tether the fusion complex to acetylated chromatin while the NUT moiety recruits/activates p300/CBP to establish an acetylation feed-forward loop and expand active chromatin domains (23), providing a unified rationale for BET and HDAC inhibitor strategies. At a higher organizational level, liquid–liquid phase separation (LLPS) condensates (24), megadomain formation (25, 26), and 3D genome reorganization collectively shape a stable transcriptional niche (22), reinforcing core oncogenic transcriptional programs and suppressing differentiation-related pathways (21, 22, 27).Consequently, a poorly differentiated/undifferentiated morphology can be viewed as an expected epigenetic outcome rather than requiring the assumption of a single tissue of origin. The enrichment of specific fusion partners across lineage- and site-stratified subsets should be interpreted as a probabilistic and hypothesis-generating tendency related to cellular context and tissue microenvironment rather than as evidence of deterministic lineage specification. Structural differences among fusion partners may alter chromatin engagement (16), cofactor recruitment, or transcriptional circuit modulation, while the cell of origin and local environment may influence which fusion events are selected and how they manifest histologically. However, for rare non-BET fusions such as *YAP1::NUTM1* and *MGA::NUTM1*, available evidence remains insufficient to establish whether the fusion directly drives the observed non-squamous differentiation states or instead marks a correlation shaped by sampling and publication bias.

### 4.4 Models and modalities

**Figure 6:**
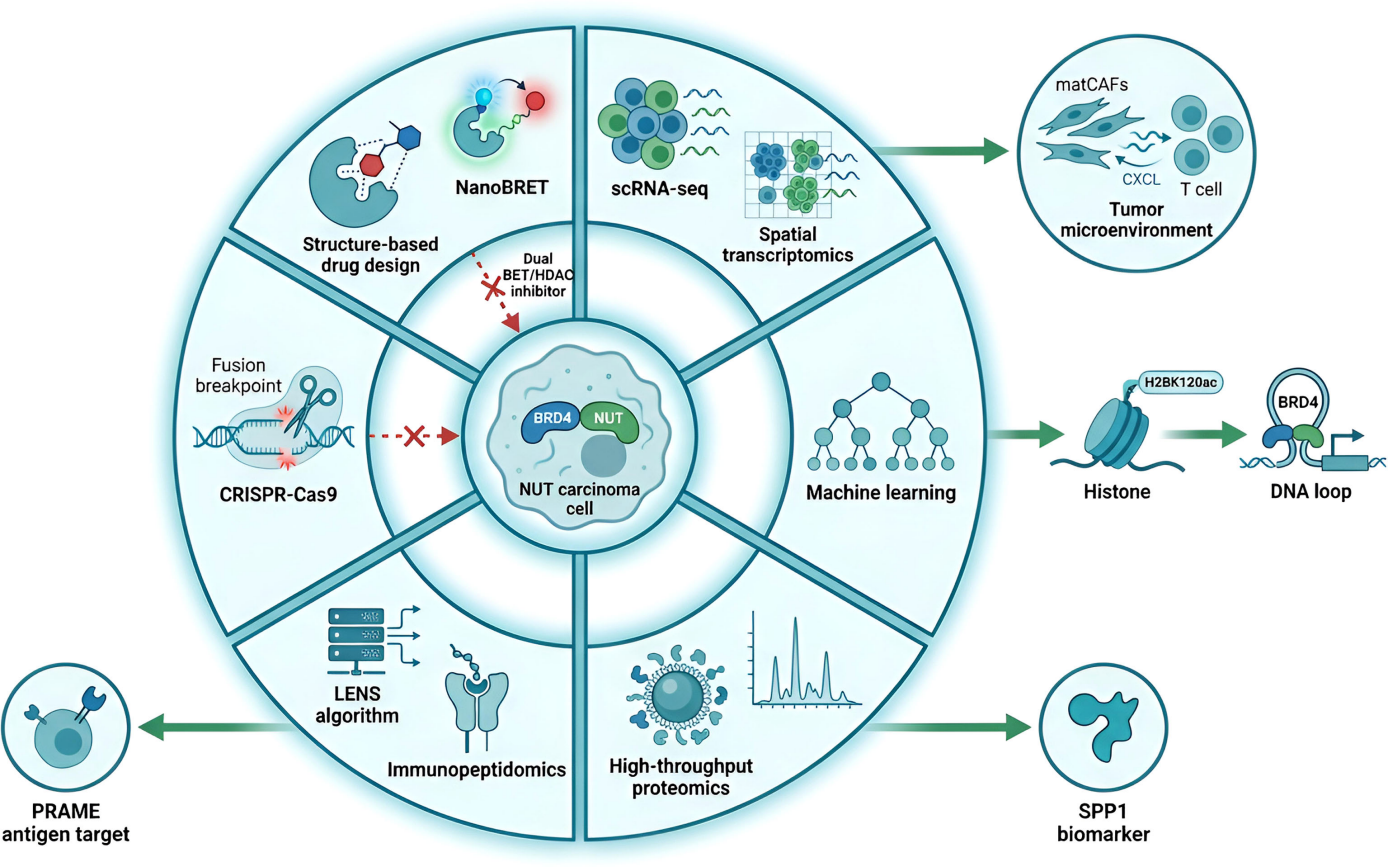
Multimodal platforms and emerging technologies for mechanistic dissection and precision translation in NUT carcinoma. scRNA-seq, single-cell RNA sequencing; BET, bromodomain and extra-terminal; HDAC, histone deacetylase.

The mechanistic framework outlined above provides an interpretable coordinate system; however, its testability and translational utility still depend on appropriate model systems and robust phenotypic readouts. Consistent with the bibliometric signal of increasing emphasis on molecular diagnosis and translation, key advances in NUT carcinoma research have been driven to a large extent by complementary model platforms and rapidly evolving multimodal technologies (**Figure 6**). Patient/mice-derived cell lines (**Supplementary Table 9**) (11, 16, 28–31)offer a foundational platform for dissecting fusion-driven chromatin remodeling and transcriptional amplification and support drug screening for epigenetic dependencies such as BET and HDAC inhibition. Inducible-expression systems and genetically engineered tagging further improve the resolution of interaction networks, chromatin states, and dynamic processes. Meanwhile, *in vivo* animal model systems—including GEMMs, knockout models, and xenografts (**Supplementary Table 10**)—provide indispensable evidence to test the oncogenic sufficiency of a single fusion driver, capture early lesions, and interrogate tumor–microenvironment interactions (20, 23, 27, 29–33).

In parallel with controllable validation enabled by model systems, multi-omics and spatial multimodal technologies further enhance resolution for tumor heterogeneity and microenvironmental remodeling. These approaches can delineate intratumoral differentiation states, invasion-associated subpopulations, and microenvironmental changes, thereby offering finer stratification cues and potential biomarkers; they can also provide trackable molecular readouts of treatment response and tolerance/resistance mechanisms to inform rational combination strategies (9, 34, 35). Proteomics and immunopeptidomics further expand the discovery space for circulating/tissue biomarkers and immunologic targets, whereas computational approaches such as machine learning may help distill key determinants from multi-omic or epigenetic features (36–39). Therefore, promoting multimodal strategies and aligning these technologies with clinical workflows may reduce under-detection and shorten time to diagnosis in the future.

Future mechanistic validation will be particularly important for variant non-BET fusions. Patient-derived cell lines, organoid or xenograft models, and genetically engineered mouse models carrying defined fusion partners could be used to test whether rare fusions directly reshape chromatin engagement, epigenetic reader–writer complex assembly, and downstream lineage programs. Such models would help distinguish fusion-driven lineage specification from statistical enrichment caused by case selection, tissue context, or publication bias.

### 4.5 Treatment strategies for NUT carcinoma

Outside immune-based strategies, management of NUT carcinoma remains anchored in aggressive local control and empiric systemic therapy. For resectable disease, early radical surgery with an R0 margin offers the best chance of durable control, with adjuvant radiotherapy and/or systemic therapy considered according to margin status, nodal involvement, and site-specific staging principles (5, 40–43).For unresectable disease or non-R0 resection, definitive high-dose IMRT-based radiotherapy combined with systemic therapy is commonly adopted, and tumor burden metrics (e.g., GTV size) may further stratify prognosis and guide intensification (10). Cytotoxic chemotherapy can induce responses but is typically limited by rapid resistance; regimen selection (e.g., platinum- vs ifosfamide-based) may influence short-term response patterns without consistently translating into long-term survival benefit (8).Epigenetic and transcriptional targeting—most notably BET inhibition, as well as HDAC inhibition and other emerging agents—provides an additional systemic option and a rational partner for multimodal combinations, albeit with practical constraints such as hematologic toxicity and limited CNS penetration in some settings (9, 34, 38, 44–49).

Consistent with the recent bibliometric shift toward immunotherapy and tumor microenvironment–related topics, immunotherapy has emerged as a practical frontier in thoracic and head and neck NUT carcinoma. To contextualize the heterogeneous clinical reports, we integrate our biomarker synthesis (PD-L1, MSI, and TMB) with a focused evidence map of ICI regimens and outcomes. As shown in **Table 2**, evidence is largely limited to case reports/series and retrospective cohorts, with no randomized data (50–69).

**Table 2:** Comprehensive summary of immunotherapy outcomes in NUT carcinoma reported in literature.

Reference (journal)	Patients n (age/sex)	Primary site	ICI regimen	Best response	Outcome	Biomarkers (PD-L1; TMB)	irAEs
Yang et al. (2025) (Front Immunol)	1(32/M)	Lung	Sintilimab + etoposide + carboplatin (1L); PD-1 re-challenge after BET inhibitor (BETi)	PR (1L); PD (re-challenge)	OS 15 months	PD-L1: TPS NR (assay NR; reported negative); TMB: NR	NR
Davis et al. (2021) (Clin Lung Cancer)	1(31/F)	Lung	Nivolumab monotherapy (1L)	SD	PFS 29 months (irRECIST); continued ~13 months post-progression	PD-L1: TPS 10% (SP263); TMB: NR	Hypothyroidism (grade 2)
Herbison et al. (2024) (J Immunother Precis Oncol)	1(27/F)	Sinonasal	Nivolumab 480 mg every 4 weeks (q4w) (post-radiotherapy, sequential); prior docetaxel/cisplatin/5-fluorouracil (TPF) induction	PR	PFS NR; duration of response (DOR) >12 months	PD-L1: TPS <1% (SP263); TMB: NR	NR
Badran et al. (2024) (Ann Med Surg)	1(24/M)	Lung	Pembrolizumab + carboplatin + paclitaxel (1L)	PD	OS NR (1L failed at 3 cycles; cord compression)	PD-L1: TPS 0% (22C3); TMB: NR	Type 1 diabetes mellitus (T1DM); diabetic ketoacidosis (DKA)
Cao et al. (2024) (Gland Surg)	1(32/F)	Thyroid	Nivolumab + nab-paclitaxel + anlotinib + radiotherapy (RT) (70 Gray [Gy])	SD	OS 7 months	PD-L1: TPS NR (assay NR; reported negative); TMB: 1.86 mutations per megabase (mut/Mb)	Hypothyroidism
Lai et al. (2024) (BMJ Case Rep)	1(Young/Pregnant)	Lung	Pembrolizumab monotherapy (2L after cisplatin/etoposide failure)	PD	OS 50 days post-admission	PD-L1: TPS 0% (assay NR); TMB: NR	NR
Haebe et al. (2025) (npj Precis Oncol)	1(41/F)	Nasopharynx	Pembrolizumab + cisplatin + paclitaxel + radiotherapy (RT) (20 Gray [Gy]/5 fractions [fx])	PR	OS 4 months (PR at 3 months; rapid PD after ICI discontinuation)	PD-L1: TPS 100% (SP263); TMB: 2.4 mut/Mb	Pneumonitis; thyroiditis (led to ICI stop)
Ugurluer et al. (2024) (Cancer Radiother)	1(17/M)	Larynx	Nivolumab 3 mg/kg every 3 weeks (q3w) + ifosfamide/carboplatin/etoposide (ICE)	PD	OS ~15 months	PD-L1: TPS/CPS NR (assay NR; reported positive); TMB: NR	NR
Cadesky A et al. (2024) (JCEM Case Rep)	1(27/F)	Thyroid	Pembrolizumab + carboplatin + paclitaxel (~3 months)	PD	OS ~5–6 months	PD-L1: TPS 100% (assay NR); TMB: NR	Adrenal insufficiency
Caner et al. (2024) (J Cancer Res Ther)	1(29/F)	Sinonasal	Pembrolizumab + cisplatin/5-fluorouracil (5-FU) (+ docetaxel after cycle 1)	PD	OS 5.4 months	PD-L1: TPS 10% (assay NR); TMB: NR	NR
Lee et al. (2023) (Cancer Res Treat)	1(45/M)	Lung	Pembrolizumab monotherapy (line NR)	NR	PFS 2 months	PD-L1: TPS NR (assay NR); TMB: NR	NR
Fu et al. (2023) (Front Oncol)	1(36/M)	Parotid	Sintilimab + lenvatinib + BET inhibitor (BETi; NHWD-870) (≥3L)	PR	OS ~25 months (PR maintained ~6 cycles)	PD-L1: TPS <1% (assay NR); TMB: 4 mut/Mb	NR
Chen et al. (2023) (J Cancer Res Clin Oncol)	2 (from n=6; 31–73 y)	Lung	Pt1: Durvalumab + paclitaxel/carboplatin (TP) (adjuvant); Pt2: Atezolizumab + etoposide/cisplatin (EP) + radiotherapy (RT) (neoadjuvant)	Pt1: PD; Pt2: pCR	Pt1: OS 4 months; Pt2: PFS 10 months; no evidence of disease (NED) at 10 months	Pt1: PD-L1 TPS 0% (assay NR); TMB: NR; Pt2: PD-L1 TPS 0% (assay NR); TMB: NR	Pt1: NR; Pt2: NR
Flaadt et al. (2024) (Pediatr Blood Cancer)	2 (from n=11; median 13.2 y)	Lung (Pt10); Cervical lymph nodes (Pt11)	Pt10: Pembrolizumab (later-line, as part of multi-agent therapy); Pt11: Pembrolizumab (2L) → pembrolizumab + talimogene laherparepvec (T-VEC) + carboplatin/etoposide (3L)	Pt10: PD; Pt11: 2L SD then PD; 3L PR then mixed response	Pt10: OS 107.5 months; Pt11: OS 30.6 months (mixed response at 3 years 5 months)	PD-L1: TPS/CPS NR (assay NR); TMB: NR	NR
Kloker et al. (2022) (Front Oncol)	1(57/F)	Lung	Pembrolizumab + talimogene laherparepvec (T-VEC) (intratumoral) + chemotherapy (vincristine/cyclophosphamide/doxorubicin/etoposide [VCDE])	PR (after cycle 2); SD (after cycle 3, slow progression)	PFS NR; ongoing at 6 months	PD-L1: TPS <1% (assay NR); TMB: Low (mut/Mb NR)	NR
Zhou et al. (2021) (Front Oncol)	1(38/M)	Thyroid	Camrelizumab + epirubicin + paclitaxel liposomes	PD	OS ~10 months	PD-L1: CPS >30 (assay NR); TMB: NR	NR
Riess et al. (2021) (Transl Oncol)	2 (from n=31; median 43 y)	Thyroid	Pt1: Nivolumab monotherapy → nivolumab + ipilimumab; Pt2: Atezolizumab + carboplatin/paclitaxel + radiotherapy (RT)	Pt1: near-complete response; Pt2: PR	Pt1: PFS 4 months (near-complete response at 2 months); Pt2: PFS 5 months	Pt1: PD-L1 TPS NR (Ventana SP142 and Dako 22C3; reported negative); TMB: 0.8 mut/Mb; Pt2: PD-L1 TPS 1% (assay NR); TMB: 1 mut/Mb	Pt1: NR; Pt2: NR
Cho et al. (2020) (Thorac Cancer)	3 (from n=10; 18–49 y)	Lung	Pt1: Pembrolizumab + carboplatin/Genexol + radiotherapy (RT); Pt2: Pembrolizumab + pemetrexed/carboplatin (Alimta/carboplatin); Pt8: Neoadjuvant carboplatin/Genexol/pembrolizumab → surgery → adjuvant pembrolizumab	Pt1: NR; Pt2: CR; Pt8: CR	Pt1: OS NR; PFS NR (transferred); Pt2: OS NR; PFS NR (CR achieved); Pt8: OS NR; PFS NR (CR achieved)	Pt1: PD-L1 TPS 80% (assay NR); TMB: NR; Pt2: PD-L1 TPS 70% (assay NR); TMB: NR; Pt8: PD-L1 TPS 0% (assay NR); TMB: NR	Pt1: NR; Pt2: NR; Pt8: NR
Costa et al. (2022) (Clin Lung Cancer)	1(33/M)	Lung	Pembrolizumab + carboplatin + nab-paclitaxel + radiotherapy (RT)	SD	OS 4.7 months (initial mild response at 2 cycles; new brain/spine metastases at 4 cycles)	PD-L1: TPS <1% (assay NR); TMB: NR	NR
Aryal et al. (2021) (Diagn Cytopathol)	1(36/F)	Lung	Pembrolizumab + carboplatin + nab-paclitaxel	NR	OS ~8 months from presentation	PD-L1: TPS NR (assay NR; reported negative); TMB: NR	NR

ICI, immune checkpoint inhibitor; OS, overall survival; PFS, progression-free survival; PR, partial response; SD, stable disease; PD, progressive disease; CR, complete response; pCR, pathologic complete response; DOR, duration of response; NED, no evidence of disease; TPS, tumor proportion score; CPS, combined positive score; PD−L1, programmed death-ligand 1; TMB, tumor mutational burden; irRECIST, immune-related RECIST; RT, radiotherapy; Gy, Gray; fx, fractions; NR, not reported. Biomarkers: PD-L1 is reported as TPS or CPS as stated in the original publication, with assay clone, scoring framework, and cutoff listed when available. No assumption of direct TPS/CPS interchangeability was made. TMB is reported as mutations per megabase (mut/Mb) when available; “Low” indicates low TMB as described by the source with numeric value not reported.Time notation: “months/years” indicate the duration from the relevant treatment start or as defined by the source; “at” denotes timepoint of assessment.Responses/outcomes: Best response follows authors’ reporting (e.g., RECIST/irRECIST when specified). When both OS and PFS are available, OS is shown; additional outcome descriptors (e.g., DOR, NED) are provided as supplementary information in the Outcome column. Safety: The irAEs column summarizes immune-related adverse events attributable to ICI therapy when reported; other treatment-related adverse events may not be captured unless explicitly described.

The immune checkpoint inhibitors (ICIs) reported to date are predominantly PD-1/PD-L1 inhibitors, including pembrolizumab, nivolumab, sintilimab, camrelizumab, durvalumab, and atezolizumab (52, 54, 55, 57–68).In terms of treatment strategy, ICIs are most often used in combination with platinum-based chemotherapy and/or radiotherapy (e.g., maintenance after concurrent/sequential chemoradiotherapy or combined with chemotherapy), suggesting that in real-world practice ICIs are typically embedded within multimodal treatment rather than used alone (52, 54, 55, 57, 59, 60, 63, 65).Beyond conventional chemoradiotherapy combinations, ICIs have also been combined (or sequenced) with BET inhibitors, anti-angiogenic/multi-target TKIs, or the oncolytic virus T-VEC in an attempt to enhance efficacy (54, 56, 59, 60, 62).

Overall efficacy is markedly heterogeneous: in primary pulmonary NUT carcinoma, there is a reported case of nivolumab monotherapy achieving sustained stable disease (SD) for approximately 29 months, with continued treatment beyond radiographic progression and ongoing clinical benefit, indicating a potential long-term benefit window in a minority of patients (50).Meanwhile, there are reports of CR/pCR achieved after perioperative or chemoradiotherapy combined with immunotherapy, suggesting that deep responses may be attainable with specific sequencing and combination patterns (54, 55).However, most cases still show primary resistance or rapid progression after only transient benefit (including early progression on first-line platinum-based chemotherapy plus PD-1 inhibitors), indicating that ICI efficacy in NUT carcinoma is unstable and may be substantially influenced by the tumor’s aggressive biology and confounding from concomitant therapies (52, 61, 64–66, 69).

From a biomarker perspective, PD-L1 expression spans a wide range in NUT carcinoma: some cases show high TPS (e.g., 70–80% and 100%), whereas others have TPS of 0% or near 0% (55, 62, 63, 66, 69).Among cases with available data, NUT carcinoma generally exhibits low TMB and is predominantly MSS; for example, a *BRD4::NUTM1* cohort reported a mean TMB of ~1.7 mut/Mb (range 0–4) with all cases MSS, suggesting that the classic “high TMB” paradigm may not apply to most NUT carcinomas (63).Notably, objective responses or substantial metabolic responses have also been observed in the setting of PD-L1 negativity and low TMB: for example, an index case with a *BRD4::NUTM1* fusion achieved near-complete response to nivolumab despite PD-L1 negativity and TMB 0.8 mut/Mb; another PD-L1–negative patient achieved pCR after adding a PD-L1 inhibitor to a chemoradiotherapy backbone (55, 63).In addition, in the context of very low PD-L1 expression (TPS <1%), multimodal therapy involving sequential T-VEC plus pembrolizumab has also shown signals such as PR/SD, suggesting that “immune-cold” tumors may be reshaped through combination strategies (54, 56).Mechanistically, a study using HLA typing and pMHC affinity prediction proposed that junctional peptides generated at the *BRD4::NUTM1* fusion breakpoint may bind with high affinity in specific HLA-C subtypes, potentially triggering effective T-cell responses even in a low-TMB context, providing a direction for future biomarker development (63).From a practical trial-design perspective, these observations suggest that PD-L1 and TMB should not be used as the only biomarker axes for future NUT carcinoma immunotherapy studies. A feasible biomarker module could include: (i) fusion breakpoint characterization by RNA-based or hybrid-capture sequencing; (ii) HLA typing from tumor or germline DNA; (iii) in silico prediction of fusion-junction peptide binding affinity to patient-specific HLA alleles; and, when tissue quantity permits, (iv) immunopeptidomic validation of presented peptides (63). This approach remains investigational and should not be interpreted as a current replacement for standard PD-L1 or MSI/TMB assessment. However, it may be particularly relevant for rare fusion-driven tumors in which conventional immunotherapy biomarkers are frequently negative or low. Recent work also suggests that PRAME may represent an additional HLA-restricted T-cell target in NUT carcinoma, supporting the broader concept that antigen-presentation-based biomarkers may complement PD-L1 and TMB in future trials (39).

Regarding safety, reported immune-related adverse events (irAEs) are typified by endocrine toxicities, including hypothyroidism/thyroiditis and suspected immune-related type 1 diabetes (which may be complicated by DKA), and immune-related pneumonitis leading to treatment discontinuation has also been described (50, 55, 66). In summary, current evidence remains limited by small sample sizes, heterogeneous study designs, confounding from combination therapies, and inconsistent endpoints/response criteria; prospective registries and integrated studies incorporating fusion breakpoints, HLA typing, pMHC affinity, and tumor microenvironment features are urgently needed to define optimal ICI combinations and the populations most likely to benefit (54, 56, 63).

### 4.6 Limitations and outlook

This study has several limitations. First, both the bibliometric analysis and case-level synthesis were based on WoSCC-indexed English-language publications. This unified source improved compatibility with citation-based bibliometric tools and ensured that bibliometric mapping and clinicopathologic synthesis were derived from the same reproducible corpus; however, it may have reduced capture of region-specific reports and rare case reports. Accordingly, pooled estimates should be interpreted as literature-derived estimates from a WoSCC-indexed English-language corpus rather than exhaustive global incidence estimates.

Second, the clinicopathologic synthesis relied predominantly on case reports and small case series, in which diagnostic timelines, immunophenotypic details, treatments, and outcomes were inconsistently reported. Endpoint-specific complete-case analyses were therefore necessary, limiting causal inference and increasing susceptibility to reporting, referral, and publication biases. Similarly, fusion partner–phenotype associations should be interpreted as statistical enrichment and hypothesis-generating signals rather than deterministic rules. This caveat is particularly important for rare fusions, for which small sample sizes and wide confidence intervals limit the stability of effect-size estimates.

Third, PD-L1 and MSI/TMB data were available only in subsets of cases and were heterogeneous with respect to assays, scoring systems, and cutoffs. Although we harmonized PD-L1 into broad categories to enable synthesis, TPS and CPS are not directly interchangeable, because TPS reflects membranous staining in tumor cells whereas CPS incorporates staining in tumor cells and selected immune cells. Therefore, three-tier PD-L1 categorization across heterogeneous literature reports may introduce misclassification and residual heterogeneity. To reduce dependence on assay-specific and cutoff-specific distinctions, we performed a simplified Negative versus Any expression sensitivity analysis, which confirmed the central finding that PD-L1 negativity predominates in reported thoracic and head and neck NUT carcinoma. Nevertheless, the small number of PD-L1-expressing tumors and the scarcity of rare fusion partners preclude definitive conclusions regarding fusion-specific immune-biomarker patterns. Although institutional PD-L1 testing was performed uniformly using the 22C3 pharmDx assay and TPS scoring, the literature-derived cases used heterogeneous assays, clones, and scoring frameworks; therefore, cross-cohort comparisons should be interpreted as broad pattern-level comparisons rather than assay-equivalent biomarker estimates.

Finally, missingness in the institutional cohort limited subgroup validation. Histologic information was unavailable in 20 of 59 patients, and rare non-BET fusions such as *YAP1::NUTM1* and *MGA::NUTM1* were not represented among fusion-annotated cases. Accordingly, the institutional cohort supports the broader signals of *BRD4::NUTM1* predominance and PD-L1 negativity, but cannot independently validate rare fusion-specific phenotype enrichment.

Future research should focus on: (1) establishing more standardized case reporting and data elements (fusion type, primary site, histologic description, immunophenotype, diagnostic timeline, etc.); (2) leveraging registries and multicenter datasets to reliably link molecular subtypes with treatment and outcomes; (3) testing, within defined cellular origin/lineage contexts, the interaction between “fusion-partner domains × cellular background” in shaping chromatin organization and transcriptional circuits; (4) prospectively evaluating workflow-embedded multimodal diagnostic strategies and mechanism-informed diagnostic and combination-therapy approaches; and (5) developing better biomarkers and rational combination strategies for predominantly immune-cold tumors. Aligning bibliometrically identified frontiers with unmet real-world clinical needs may help accelerate the translation of NUT carcinoma from “recognition” to “reproducible diagnosis and effective therapy”.

## 5 Conclusion

This study integrates bibliometric analysis with systematic clinicopathologic synthesis to characterize thoracic and head-and-neck NUT carcinoma. We identified non-random fusion-partner distributions and a predominantly PD-L1-negative immune-biomarker profile. The independent institutional cohort supported the broader literature-derived signals of BRD4::NUTM1 predominance and PD-L1 negativity, but was not powered to validate rare fusion-specific enrichment patterns. Exploratory fusion partner–phenotype enrichment patterns may refine diagnostic suspicion and guide future mechanistic research, but require validation in larger multicenter cohorts and dedicated model systems. Overall, these findings highlight the clinical value of integrating fusion architecture with immune profiling and support prospective biomarker-integrated studies in NUT carcinoma.

## Data Availability

The original contributions presented in the study are included in the article/**Supplementary Material**. Further inquiries can be directed to the corresponding authors.
