# Supplementary material for: Immune biomarker landscape and fusion partner–phenotype associations in thoracic and head-and-neck NUT carcinoma
Source: Front Immunol. 2026 May 28;17:1834366. doi: 10.3389/fimmu.2026.1834366 (PMC13253808; doi:10.3389/fimmu.2026.1834366)

**Contents**

# Supplementary Table S1. Literature search strategy

| **#** | **Search Query** | **Database** | **Results** | **Date Run** |
| --- | --- | --- | --- | --- |
| 1 | (TS=(NMC) OR TS=(NUTM1 fusion) OR TS=(NUTM1-rearranged) OR TS=(BRD4) OR TS=(BRD3) OR TS=(NSD3) OR TS=(ZNF532) OR TS=(ZNF592)) | Web of Science Core Collection | 10353 | Sun Jan 04 2026 11:27:37 GMT+0800 |
| 2 | TS=(CANCER) OR TS=(carcinoma) OR TS=(Neoplasm) | Web of Science Core Collection | 4256706 | Sun Jan 04 2026 11:28:02 GMT+0800 |
| 3 | TS=("NUT carcinoma" OR "NUT midline carcinoma" OR "NUTM1-rearranged carcinoma" OR "NUTM1 fusion" OR "NUTM1-rearranged squamous cell" OR "NUT rearrangement" OR "Nuclear protein in testis" OR "Nuclear protein of the testis midline" OR "NUTM1 protein") | Web of Science Core Collection | 623 | Sun Jan 04 2026 11:28:20 GMT+0800 |
| 4 | #3 OR #1 | Web of Science Core Collection | 10666 | Sun Jan 04 2026 11:30:43 GMT+0800 |
| 5 | #4 AND #2 | Web of Science Core Collection | 2773 | Sun Jan 04 2026 11:30:51 GMT+0800 |
| 6 | TS=(thorac* OR chest OR "chest wall" OR intrathorac* OR transthorac* OR lung* OR pulmonary OR bronch* OR trache* OR airway* OR "respiratory tract" OR pleura* OR pleural OR "pleural cavity" OR mediastin* OR thym* OR "anterior mediastinum" OR "posterior mediastinum" OR pericard* OR "superior vena cava" OR "SVC" OR esophag* OR oesophag* OR diaphrag* OR rib* OR sternum OR clavicl*) | Web of Science Core Collection | 3467866 | Sun Jan 04 2026 11:32:34 GMT+0800 |
| 7 | TS=("head and neck" OR "head & neck" OR "upper aerodigestive tract" OR UADT OR "head and neck cancer" OR "head and neck carcinoma" OR HNSCC OR sinonasal OR "sino-nasal" OR nose OR nasal OR "nasal cavity" OR paranasal OR "paranasal sinus*" OR sinus* OR maxillar* OR ethmoid* OR sphenoid* OR frontal* OR nasopharyn* OR oropharyn* OR hypopharyn* OR pharyn* OR "upper pharyn*" OR laryn* OR supraglott* OR glott* OR subglott* OR epiglott* OR "vocal cord*" OR "true vocal cord*" OR "false vocal cord*" OR oral OR "oral cavity" OR mouth OR buccal OR gingiva* OR gum* OR tongue OR "floor of mouth" OR palate OR "hard palate" OR "soft palate" OR tonsil* OR uvula OR lip* OR labial OR salivar* OR "salivary gland*" OR parotid OR submandibular OR "submandibular gland" OR "sublingual gland" OR "minor salivary gland*" OR "major salivary gland*" OR neck OR cervical OR "cervical lymph node*" OR "neck mass" OR thyroid* OR "thyroid gland" OR parathyroid* OR "parathyroid gland" OR cricoid* OR "thyroid cartilage" OR hyoid* OR "Eustachian tube" OR otic OR ear OR auricular OR "external auditory canal" OR "temporal bone" OR "skull base") | Web of Science Core Collection | 4794470 | Sun Jan 04 2026 11:32:52 GMT+0800 |
| 8 | #7 OR #6 | Web of Science Core Collection | 7808597 | Sun Jan 04 2026 11:33:01 GMT+0800 |
| 9 | #8 AND #5 | Web of Science Core Collection | 919 | Sun Jan 04 2026 11:33:07 GMT+0800 |

**Timespan: 1991–2025; Document types: Article and Review.**

# Supplementary Table S2. Agency for Healthcare Research and Quality assessment of the included studies

| **Literature** | **Sample(s)** | **Q1** | **Q2** | **Q3** | **Q4** | **Q5** | **Q6** | **Q7** | **Q8** | **Q9** | **Q10** | **Q11** | **Evidence level** |
| --- | --- | --- | --- | --- | --- | --- | --- | --- | --- | --- | --- | --- | --- |
| YAP1-NUTM1 gene fusion in porocarcinoma of the external auditory canal | 2 | Y | N | N | N | U | U | U | U | U | U | Y | 4 |
| Uncommon somatic mutations in metastatic nut midline carcinoma | 1 | Y | N | N | N | U | U | U | U | U | U | Y | 4 |
| Tumor Mutation Burden and Checkpoint Immunotherapy Markers in NUT Midline Carcinoma | 2 | Y | Y | Y | Y | Y | Y | Y | Y | Y | Y | Y | 4 |
| Thyroid Carcinoma with NSD3::NUTM1 Fusion: a Case with Thyrocyte  Differentiation and Colloid Production | 1 | Y | N | N | N | N | U | U | N | N | N | U | 4 |
| Thyroid Carcinoma With NSD3::NUTM1 Fusion and Secondary TERT Promoter Mutation: A Case Report and Literature Review | 2 | Y | N | Y | N | Y | Y | N | N | N | N | Y | 4 |
| Thoracic NUT carcinoma: expanded pathologic spectrum with expression of TTF-1 and neuroendocrine  markers | 6 | Y | N | Y | Y | U | U | Y | N | N | U | Y | 4 |
| Thoracic NUT carcinoma: Common pathological features despite diversity  of clinical presentations | 1 | Y | N | N | N | U | U | U | N | N | U | Y | 4 |
| Thoracic nuclear protein in testis (NUT) carcinoma: Expanded pathological spectrum with expression of thyroid transcription factor-1 and neuroendocrine markers | 3 | Y | U | Y | Y | N | N | N | N | N | N | U | 4 |
| The first report of molecular characterized BRD4-NUT carcinoma in Brazil: a case report. | 1 | Y | N | N | N | U | U | U | U | U | U | Y | 4 |
| Sustained Clinical Response to Immunotherapy Followed by BET Inhibitor in a Patient with Unresectable Sinonasal NUT Carcinoma | 1 | Y | N | Y | N | Y | Y | N | N | N | N | Y | 4 |
| Skin‑analogue primary poroid neoplasms of the head and neck  with YAP1/WWTR1::MAML2/NUTM1 fusions: clinicopathologic  and genetic spectrum of a novel tumor family delineated in a series  of 10 cases | 7 | Y | N | Y | N | Y | Y | N | N | N | N | Y | 4 |
| Sinonasal nut-midline carcinoma - a multimodality approach to diagnosis, staging and post-surgical restaging | 1 | Y | N | U | N | U | U | N | U | U | U | Y | 4 |
| Sinonasal NUT carcinoma A retrospective case series from a single institution | 1 | Y | N | Y | N | N | N | N | N | N | N | Y | 4 |
| Sinonasal Differentiated Papillary NUT Carcinoma | 1 | Y | Y | Y | Y | Y | Y | Y | Y | Y | Y | Y | 4 |
| Sarcoma with MGA::NUTM1 fusion: a report of three cases and literature review | 1 | Y | N | Y | U | N | N | N | N | N | N | Y | 4 |
| Sarcoma with MGA-NUTM1 fusion in the lung: an emerging entity | 1 | Y | N | U | U | U | U | U | U | U | U | Y | 4 |
| Salivary Gland NUT Carcinoma with Prolonged Survival in Children:  Case Illustration and Systematic Review of Literature | 1 | Y | N | N | N | U | U | U | U | N | N | Y | 5 |
| Pulmonary NUT carcinoma, an elusive and refractory entity, shows transient response to chemotherapeutics and PD-1 inhibitor: a case report and literature review | 1 | Y | N | N | U | U | U | U | N | N | N | Y | 4 |
| Pulmonary Nuclear protein in Testis (NUT) carcinoma: clinical, molecular characteristics, and treatment strategies | 3 | Y | U | Y | Y | N | N | N | N | N | N | U | 4 |
| Prolonged Survival of NUT Midline Carcinoma and Current  Approaches to Treatment | 1 | Y | N | Y | N | Y | Y | N | N | N | N | Y | 4 |
| Primary Thyroid NUT Carcinoma With High PD-L1 Expression and Novel Massive IGKV Gene Fusions: A Case Report With Treatment Implications and Literature Review | 1 | Y | N | N | N | N | N | N | N | N | N | N | 4 |
| Primary thyroid nuclear protein in testis carcinoma: a case report  and literature review | 1 | Y | N | Y | N | Y | Y | N | N | N | N | Y | 4 |
| Primary spindle cell sarcoma of the lung with MGA::NUTM1 fusion: an extremely rare case of a potentially emerging entity and review of the literature | 1 | Y | N | N | N | N | N | N | N | N | N | N | 4 |
| Primary pulmonary NUT midline carcinoma-clinical radiographic and pathologic characterization | 8 | Y | U | Y | U | U | U | N | N | N | N | Y | 4 |
| Primary Pulmonary NUT Carcinoma: Case Illustration and Updated Review of Literature | 1 | Y | Y | Y | Y | Y | Y | Y | Y | Y | Y | Y | 4 |
| Primary Pulmonary NUT Carcinoma: An Aggressive and Rare Tumor | 2 | Y | Y | Y | Y | Y | Y | Y | Y | Y | Y | Y | 4 |
| Primary Cutaneous NUT Carcinoma Clinicopathologic and Genetic Study of 4 Cases | 2 | Y | Y | Y | Y | Y | Y | Y | Y | Y | Y | Y | 4 |
| Possible Primary Thyroid Nuclear Protein in Testis Carcinomas with NSD3::NUTM1 Translocation Revealed by RNA Sequencing: A Report of Two Cases | 1 | Y | N | N | N | U | N | N | N | N | U | Y | 4 |
| Poroid adnexal skin tumors with YAP1 fusions exhibit similar histopathologic features: A series of six YAP1-rearranged adnexal skin tumors | 1 | Y | N | N | N | U | N | N | N | N | U | U | 4 |
| Placental metastasis from maternal NUT carcinoma:  diagnostic pitfalls and challenges | 1 | Y | Y | Y | Y | Y | Y | Y | Y | Y | Y | Y | 4 |
| Pediatric NUT carcinoma: a patient series and literature review | 2 | Y | N | N | N | U | N | N | N | N | N | N | 4 |
| Pathologic characteristics of NUT midline carcinoma arising in the mediastinum | 1 | Y | N | Y | N | N | N | N | N | N | N | N | 4 |
| Oral and oropharyngeal NUT carcinoma: a multicentre screening study of poorly differentiated oral cancer | 3 | Y | Y | Y | Y | Y | Y | Y | Y | Y | N | Y | 4 |
| NUTM1-rearranged neoplasia: a multiinstitution experience yields novel fusion partners and expands the histologic spectrum | 21 | Y | N | U | U | U | U | U | U | U | Y | Y | 4 |
| NUTM1 gene fusions characterize a subset of undifferentiated soft tissue and visceral tumors | 1 | Y | N | Y | U | U | U | N | U | U | U | Y | 4 |
| NUTM1 -rearranged Carcinoma of the Thyroid : A Distinct Subset of NUT Carcinoma Characterized by Frequent NSD3 - NUTM1 Fusions. | 9 | Y | N | Y | Y | N | U | U | N | N | N | N | 4 |
| Nut midline lung cancer: a rare case report with literature review | 1 | Y | N | N | N | N | U | N | N | N | U | Y | 5 |
| NUT midline carcinoma: Report of 2 cases suggestive of pulmonary origin | 2 | Y | N | N | U | U | U | U | N | N | N | Y | 4 |
| NUT midline carcinoma: case report and review of the literature | 1 | Y | N | N | N | U | N | N | N | N | N | N | 4 |
| NUT midline carcinoma: a series of five cases | 1 | Y | N | Y | U | U | U | N | N | N | N | N | 4 |
| NUT midline carcinoma: a neoplasm with diagnostic challenges in cytology | 1 | Y | N | N | N | U | U | U | U | N | N | Y | 4 |
| NUT midline carcinoma: a case report with a novel translocation and review of the literature | 1 | Y | N | N | N | U | U | U | U | U | U | Y | 4 |
| NUT midline carcinoma of the parotid gland with  mesenchymal differentiation | 1 | Y | N | N | N | U | U | U | U | N | N | Y | 4 |
| NUT midline carcinoma of the nasal cavity | 1 | Y | N | N | N | N | N | N | N | N | N | Y | 4 |
| NUT midline carcinoma of the mediastinum showing two types of poorly differentiated tumor cells: A case report and a literature review | 1 | Y | N | N | N | U | U | U | U | U | U | Y | 5 |
| NUT midline carcinoma mimicking a germ cell tumor: a case report | 1 | Y | N | N | N | U | U | U | U | N | N | Y | 4 |
| NUT Midline Carcinoma An Aggressive Intrathoracic Neoplasm | 1 | Y | N | N | N | N | N | N | N | N | N | Y | 4 |
| Nut midline carcinoma mimicking tonsillitis in an eight-year-old girl | 1 | Y | N | N | N | N | U | U | U | U | U | Y | 4 |
| NUT carcinoma: An uncommon, yet the overlooked entity | 1 | Y | N | Y | N | N | N | N | N | N | N | N | 4 |
| NUT Carcinoma: A Clinical, Morphological and Immunohistochemical Mimicker—The Role of RNA Sequencing in the Diagnostic Procedure | 2 | Y | N | N | N | N | N | N | N | N | N | Y | 4 |
| NUT carcinoma, an under-recognized malignancy: a clinicopathologic and molecular series of 6 cases showing a subset of patients with better prognosis and a rare ZNF532::NUTM1 fusion | 5 | Y | N | U | N | U | U | U | U | U | U | Y | 4 |
| Nut carcinoma without upfront surgical resection: a case report | 1 | Y | N | N | U | U | U | U | N | N | N | Y | 4 |
| NUT carcinoma of the parotid gland: report of two cases, one  with a rare ZNF532‑NUTM1 fusion | 1 | Y | N | Y | Y | U | U | U | U | U | U | Y | 4 |
| NUT carcinoma of the thyroid:  an unusual case with a complete response to treatment. | 1 | Y | N | N | N | U | U | U | U | U | U | Y | 4 |
| NUT carcinoma of the salivary glands: clinicopathologic and molecular  analysis of 3 cases and a survey of NUT expression in salivary  gland carcinomas | 2 | Y | N | N | N | U | U | U | U | N | N | Y | 4 |
| NUT Carcinoma of the Head and Neck: Clinicopathologic and  Molecular Analysis of 18 Cases | 14 | Y | N | N | U | N | N | N | N | N | N | Y | 4 |
| NUT Carcinoma in a Patient with Unusually Long Survival and False  Negative FISH Results | 1 | Y | N | N | N | U | U | U | U | N | N | Y | 4 |
| NUT carcinoma in a nutshell: a diagnosis to be considered more frequently | 1 | Y | N | N | N | N | N | N | N | N | N | Y | 4 |
| NUT  carcinoma in children and adults: a multicenter retrospective  study | 8 | Y | U | N | Y | N | U | U | U | U | U | Y | 4 |
| Nuclear protein in testis midline carcinomas: a le- thal and underrecognized entity | 1 | Y | N | N | N | U | U | U | U | N | N | Y | 4 |
| Nuclear protein in testis midline carcinoma with unusual elevation of α-fetoprotein and synaptophysin positivity: A case report and review of the literature | 1 | Y | N | N | U | U | U | U | N | N | N | Y | 4 |
| Nuclear protein in testis (NUT) midline carcinoma of the larynx: A rare case report of a paediatric patient and literature review | 1 | Y | Y | Y | Y | Y | Y | Y | Y | Y | Y | Y | 4 |
| NSD3::NUTM1 Fusion Sarcoma Mimicking Malignant Peripheral Nerve Sheath Tumor with Prolonged Survival | 1 | Y | N | Y | N | Y | Y | N | N | N | N | Y | 4 |
| NSD3::NUTM1 fusion evidenced on RNA sequencing in poorly differentiated thyroid cancer: a report of two cases | 2 | Y | N | N | N | U | U | U | U | U | U | Y | 4 |
| NSD3-NUTM1-rearranged carcinoma of the  median neck/thyroid bed developing after recent thyroidectomy for  sclerosing mucoepidermoid carcinoma with eosinophilia: report  of an extraordinary case | 1 | Y | N | Y | N | Y | Y | N | N | N | N | Y | 4 |
| NSD3-NUT-expressing midline carcinoma of the lung: first characterization of primary cancer tissue | 1 | Y | N | N | N | N | N | N | N | N | N | Y | 4 |
| Narrative review: this or that?-uncommon challenges in mediastinal pathology | 1 | Y | N | N | N | U | U | U | U | U | U | N | 4 |
| Molecular analysis of NUT-positive poromas and porocarcinomas identifies novel break points of YAP1::NUTM1 fusions | 3 | Y | N | N | N | U | N | N | N | N | U | U | 4 |
| Mediastinal NUT Carcinoma With Raised Serum Alpha-Fetoprotein Mimicking a Malignant Germ Cell Tumor: Suspicion Raised Due to Negative Serum miR-371a-3p Levels | 1 | Y | N | N | N | N | N | N | N | N | N | N | 4 |
| Magnifying Glass on Sinonasal NUT Carcinoma Heterogeneity via Spatial Transcriptomics | 3 | Y | N | N | N | U | N | N | N | N | U | U | 4 |
| Intra‑, para‑, and suprasellar nuclear protein of testis carcinoma with infiltration of cavernous sinus and clivus—a case report | 1 | Y | N | N | N | N | N | N | N | N | N | Y | 4 |
| Immunodetection of NUT Protein: Implementation, Indications, and Results in a Tertiary Reference Center | 5 | Y | Y | Y | Y | Y | Y | Y | Y | Y | N | Y | 4 |
| Genomic profiling of solid tumors harboring BRD4-NUT and response to immune checkpoint inhibitors | 2 | Y | N | N | U | U | U | U | N | N | N | U | 4 |
| First evidence of treatment efficacy in metastatic carcinoma of  the parotid gland with BRD4/NUT translocation | 1 | Y | N | N | N | U | U | U | U | N | N | Y | 4 |
| Differentiation of NUT midline carcinoma by epigenomic reprogramming | 1 | Y | N | N | N | N | N | N | N | N | N | Y | 4 |
| Differentiated Papillary NUT Carcinoma: An Unexpected, Deceptively Bland Presentation of a Sinonasal Carcinoma | 1 | Y | N | N | N | N | N | N | N | N | N | Y | 4 |
| Diagnosis of NUT Carcinoma Despite  False-Negative Next-Generation Sequencing  Results: A Case Report and Literature Review | 1 | Y | N | N | N | U | U | U | U | U | U | Y | 4 |
| Detecting disease-defining gene fusions in unclassified round cell sarcomas using anchored multiplex PCR/targeted RNA nextgeneration sequencing-molecular and clinicopathological characterization of 16 cases | 2 | Y | N | N | N | N | N | N | N | N | N | Y | 4 |
| Cytopathologic features of NUT midline carcinoma: a series of 26 specimens from 13 patients. | 8 | Y | N | Y | U | U | U | Y | N | N | U | U | 4 |
| Cytological features of NUT-carcinoma harbouring an  NSD3-NUTM1 fusion | 1 | N | N | N | N | U | U | U | N | N | U | Y | 4 |
| Cytological features of NUT midline carcinoma arising  in sino-nasal tract and parotid gland: report of two new cases and  review of the literature | 2 | Y | N | N | N | N | U | N | N | N | N | Y | 4 |
| Cytological features of a variant NUT midline carcinoma of the lung harboring the NSD3-NUT fusion gene: a case report and literature review | 1 | Y | N | N | N | N | U | N | N | N | N | Y | 4 |
| Comprehensive genetic profiling of six  pulmonary NUT carcinomas with a novel micropapillary histological  subtype in two cases | 4 | Y | N | Y | U | U | U | N | U | N | N | Y | 4 |
| Clinicopathological and Preclinical Findings of NUT Carcinoma: A Multicenter Study | 4 | Y | N | Y | Y | U | U | U | U | U | U | Y | 4 |
| Clinical, Radiographic, Pathologic Characterization and Survival Outcomes of Nuclear Protein of the Testis Carcinoma | 2 | Y | U | Y | Y | U | U | U | U | U | U | Y | 4 |
| Clinical Response of Carcinomas Harboring the BRD4–NUT Oncoprotein to the Targeted Bromodomain Inhibitor OTX015/MK-8628 | 2 | Y | N | Y | Y | U | U | U | U | U | U | Y | 4 |
| Clinical and molecular features of pulmonary NUT carcinoma  characterizes diverse responses to immunotherapy, with a pathologic  complete response case | 5 | Y | N | Y | N | U | U | U | U | U | U | Y | 4 |
| Challenging diagnosis in pulmonary nut carcinoma: A report of two cases with different histopathologic and molecular features and a novel NUTM1::SPECC1 gene fusion | 2 | Y | N | N | N | N | U | N | N | N | N | Y | 4 |
| Challenging Diagnosis in NUT Carcinoma | 2 | Y | N | N | N | N | N | N | N | N | N | N | 4 |
| Case report: NUT carcinoma in an elderly woman with unique morphology and immunophenotype highlights a diagnostic pitfall | 1 | Y | N | N | N | U | U | U | U | U | U | Y | 4 |
| Case report: Immunovirotherapy as a novel add-on treatment in a patient with thoracic NUT carcinoma | 1 | Y | N | N | N | U | U | U | U | U | U | Y | 4 |
| Case report of a pan-cytokeratin negative NUT midline carcinoma of pulmonary origin, a BRD3-NUT variant: Challenges in cytomorphologic presentation | 1 | Y | N | N | N | N | N | N | N | N | N | Y | 4 |
| Case Report and Literature Review: Primary Pulmonary NUT-Midline Carcinoma. | 1 | Y | N | N | N | N | N | N | N | N | N | Y | 4 |
| Cancer-Specific Sequences in the Diagnosis and Treatment of NUT Carcinoma | 9 | Y | N | Y | N | N | N | N | U | N | N | Y | 4 |
| BRD3-NUTM1-expressing NUT carcinoma of lung on  endobronchial ultr onchial ultrasound-guided tr asound-guided transbronchial needle aspir onchial needle aspiration  cytology, a diagnostic pitfall | 1 | Y | N | N | N | N | N | N | N | N | N | Y | 4 |
| Alpha-fetoprotein elevation in NUT midline carcinoma: a case report | 1 | Y | N | N | N | U | U | U | U | U | U | N | 4 |
| Adding checkpoint inhibitors to first-line chemotherapy for NUT carcinoma patients | 1 | Y | N | N | N | U | U | U | N | N | U | Y | 4 |
| A tough NUT to crack: a 47-year-old with diplopia from a rare malignancy | 1 | Y | N | N | N | N | N | N | N | N | N | Y | 4 |
| A tough NUT carcinoma to crack | 1 | Y | N | N | N | N | U | N | N | N | U | Y | 5 |
| A recurrent novel MGA-NUTM1 fusion identifies a new subtype of high-grade spindle cell sarcoma | 1 | N | N | N | U | U | U | N | U | U | U | Y | 4 |
| A rare case of NUT midline carcinoma | 1 | Y | N | N | N | U | U | U | U | N | N | Y | 4 |
| A Rare Case of NUT Carcinoma of the Thyroid | 1 | Y | Y | Y | Y | Y | Y | Y | Y | Y | Y | Y | 4 |
| A NUT carcinoma lacking squamous differentiation and expressing TTF1 | 1 | Y | N | N | N | N | N | N | N | N | N | N | 5 |
| A case of NUT midline carcinoma with complete response to gemcitabine following cisplatin and docetaxel | 1 | Y | N | N | N | U | U | N | N | N | U | Y | 4 |

# Supplementary Table S3.Top 15 Keywords with the Strongest Citation Bursts in NUT Carcinoma Research

| **Keywords** | **Year** | **Strength** | **Begin** | **End** | **1991 - 2025** |
| --- | --- | --- | --- | --- | --- |
| thymic carcinoma | 2003 | 4.82 | **2003** | 2013 | ▂▂▂▂▂▂▂▂▂▂▂▂▃▃▃▃▃▃▃▃▃▃▃▂▂▂▂▂▂▂▂▂▂▂▂ |
| aggressive carcinoma | 2004 | 6.83 | **2004** | 2013 | ▂▂▂▂▂▂▂▂▂▂▂▂▂▃▃▃▃▃▃▃▃▃▃▂▂▂▂▂▂▂▂▂▂▂▂ |
| translocation | 2004 | 5.49 | **2004** | 2013 | ▂▂▂▂▂▂▂▂▂▂▂▂▂▃▃▃▃▃▃▃▃▃▃▂▂▂▂▂▂▂▂▂▂▂▂ |
| BRD4 (bromodomain protein) | 2004 | 3.19 | **2004** | 2015 | ▂▂▂▂▂▂▂▂▂▂▂▂▂▃▃▃▃▃▃▃▃▃▃▃▃▂▂▂▂▂▂▂▂▂▂ |
| rearrangement | 2008 | 7.61 | **2008** | 2017 | ▂▂▂▂▂▂▂▂▂▂▂▂▂▂▂▂▂▃▃▃▃▃▃▃▃▃▃▂▂▂▂▂▂▂▂ |
| *BRD4::NUTM1* fusion oncogene | 2008 | 4.29 | **2008** | 2012 | ▂▂▂▂▂▂▂▂▂▂▂▂▂▂▂▂▂▃▃▃▃▃▂▂▂▂▂▂▂▂▂▂▂▂▂ |
| upper aerodigestive tract | 2009 | 3.8 | **2009** | 2012 | ▂▂▂▂▂▂▂▂▂▂▂▂▂▂▂▂▂▂▃▃▃▃▂▂▂▂▂▂▂▂▂▂▂▂▂ |
| young patients | 2009 | 3.37 | **2009** | 2013 | ▂▂▂▂▂▂▂▂▂▂▂▂▂▂▂▂▂▂▃▃▃▃▃▂▂▂▂▂▂▂▂▂▂▂▂ |
| mechanism | 2015 | 4.54 | **2015** | 2018 | ▂▂▂▂▂▂▂▂▂▂▂▂▂▂▂▂▂▂▂▂▂▂▂▂▃▃▃▃▂▂▂▂▂▂▂ |
| clinicopathological features | 2015 | 2.75 | **2015** | 2016 | ▂▂▂▂▂▂▂▂▂▂▂▂▂▂▂▂▂▂▂▂▂▂▂▂▃▃▂▂▂▂▂▂▂▂▂ |
| squamous cell carcinoma | 2008 | 2.86 | **2018** | 2021 | ▂▂▂▂▂▂▂▂▂▂▂▂▂▂▂▂▂▂▂▂▂▂▂▂▂▂▂▃▃▃▃▂▂▂▂ |
| bromodomain inhibitor(s) | 2016 | 2.41 | **2020** | 2023 | ▂▂▂▂▂▂▂▂▂▂▂▂▂▂▂▂▂▂▂▂▂▂▂▂▂▂▂▂▂▃▃▃▃▂▂ |
| head and neck | 2008 | 4.01 | **2021** | 2022 | ▂▂▂▂▂▂▂▂▂▂▂▂▂▂▂▂▂▂▂▂▂▂▂▂▂▂▂▂▂▂▃▃▂▂▂ |
| next-generation sequencing (NGS) | 2021 | 2.32 | **2021** | 2022 | ▂▂▂▂▂▂▂▂▂▂▂▂▂▂▂▂▂▂▂▂▂▂▂▂▂▂▂▂▂▂▃▃▂▂▂ |
| NUT carcinoma | 2014 | 11.96 | **2022** | 2025 | ▂▂▂▂▂▂▂▂▂▂▂▂▂▂▂▂▂▂▂▂▂▂▂▂▂▂▂▂▂▂▂▃▃▃▃ |

# Supplementary Table S4.Keyword co-occurrence clusters and LLR (log-likelihood ratio) label terms

| **Top Terms(LLR)** | **Top Terms**  **(log-likelihood ratio, p-lewel)** |
| --- | --- |
| fusion transcript; mucoepidermoid carcinoma; adenoid cystic carcinoma; nut midline carcinoma; t(11;9) mect1-maml2; t(6;9) myb-nfib; t(15;19) brd4-nut; translocation;oncogene; review | fusion transcript (8.8, 0.005); t(6 (8.8, 0.005); mucoepidermoid carcinoma (8.8, 0.005); adenoid cystic carcinoma (8.8, 0.005); t(11 (8.8, 0.005) |
| squamous cell carcinoma;mandibular neo-plasms; biphenotypic sinonasal sarcoma; sinonasal type \| paranasal sinuses; biphenotypic sinonasal sarcoma; squamous cell; sinonasal papilloma; neuroectodermal tumors | 19) squamous cell carcinoma (8.64, 0.01);sinonasal tract (8.11, 0.005); paranasal sinuses (8.11, 0.005); translocation (6.95, 0.005); sinonasal undifferentiated carcinoma (6.49, 0.05) |
| clinical imaging;european reference network; computed tomography; testis carcinoma; vdc-ie regimen \| computed tomography; lung neoplasms; nuclear protein; positron emission tomography | clinical imaging (7.15, 0.01); thyroid carcinoma (7.15, 0.01); braf (7.15, 0.01); rna polymerase ii (7.15, 0.01); nsd3 (7.15, 0.01) |
| oral squamous cell carcinoma; nut carcinoma; ovarian metastasis; parotid gland; whole-exome sequencing \| systematic review; case report; sinonasal nut carcinoma; endoscopic sinus surgery; sinonasal cancer | oral squamous cell carcinoma (8.11, 0.005); computed tomography (8.11, 0.005); rare diseases (5.89, 0.05); fdg-pet (5.89, 0.05); lung neoplasm (5.89, 0.05) |
| lung cancer; nut carcinoma; large cell carcinoma; lymphoepithetioma-like carcinoma; case report \| smarca4; lung; genetics; mediastinum; thoracic | lung cancer (11.04, 0.001); smarca4 (9.02, 0.005); who classification (9.02, 0.005); large cell carcinoma (9.02, 0.005); immune checkpoint (6.35, 0.05) |
| rna sequencing;nut carcinoma; leukocyte common antigen;expression; transcription \| expression; stem like cells; transcription; survival; fusion | rna sequencing (8.66, 0.005); expression (6.17, 0.05); r package (6.17, 0.05); insm1 (6.17, 0.05); egcg (6.17, 0.05) |
| translocation;lung cancer; carcinoma; mice; overexpression \| nuclear protein; nut-midline carcinoma; genomic analysis; bromodomain-containing protein; brd4 nut fusion | translocation (9, 0.005); laryngeal carcinoma (8.83, 0.005); bromodomain protein (8.39, 0.005); thymic carcinoma (8.39, 0.005); nut carcinoma (6.12, 0.05) |
| nuclear protein; nut midline carcinoma; ewing sarcoma; endobronchial tumour; p tefb \| acetylation; phase separation; hyperacetylation; chromatin; domain | nuclear protein (11.67, 0.001); gene expression (11.67, 0.001); super enhancers (11.67, 0.001); ovarian cancer (5.82, 0.05); domain (5.82, 0.05) |
| case report; nut carcinoma; sinonasal nut carcinoma; systematic review; midline carcinoma \| nuclear protein; genomic analysis; nut-midline carcinoma; bromodomain-containing protein; lung cancer | case report (16.36, 1.0E-4); thyroid cancer (7.63, 0.01); nuclear protein in testis (6.7, 0.01); t(15 (4.85, 0.05); carcinoma (4.53, 0.05) |
| bet inhibitor; nut carcinoma; hdac inhibitor; myc-targeting agents; treatment outcome \| nut midline carcinoma; complex genomic rearrangement; mutational signature; extra terminal; leukemia | bet inhibitor (13.05, 0.001);proteins (13.05, 0.001); bet (8.79, 0.005); resistance (8.79, 0.005); family (8.69, 0.005) |
| nut carcinoma; nuclear protein; containing 4-nuclear protein; neck cancer; containing 3-nuclear protein \| nut carcinoma; lung carcinoma; rare tumors; pd-1 inhibitor; brd4 nut fusion | nut carcinoma (7.25, 0.01); next-generation sequencing (6.58, 0.05); nut rearrangement (4.59, 0.05); undifferentiated carcinoma (4.59, 0.05); diagnosis (4.38, 0.05) |

1. Supplementary Table S5. High-Frequency Pathological Features Analysis of Thoracic and Head-Neck NUT Carcinoma

| **Category** | **Feature** | **Thoracic Frequency**  **(114 cases)** | **Head-Neck Frequency**  **(115 cases)** | **Representative Descriptions** | **Pathological Significance/Molecular Association** |
| --- | --- | --- | --- | --- | --- |
| Microscopic Patterns | Sheet-like | 38 cases | 76 cases | Dense sheet-like growth "Tumor islands arranged in sheets" | Indicates clonal proliferation and invasive growth, commonly seen in undifferentiated carcinoma |
| Microscopic Patterns | Nested | 32 cases | 54 cases | Nested and pseudopapillary arrangement "Basaloid cell nests" | Reflects partial preservation of tissue architecture, potentially retaining differentiation characteristics |
| Microscopic Patterns | Diffuse | 18 cases | 46 cases | Diffuse sheet-like growth "Diffuse infiltration into the stroma" | Suggests loss of tissue architecture and an infiltrative growth pattern |
| Microscopic Patterns | Solid | 17 cases | 41 cases | Solid growth with fibrous stroma "Dense solid clusters" | Reflects compact tumor cell proliferation, often with central necrosis, indicating high proliferative activity |
| Microscopic Patterns | Pseudopapillary | 10 cases | - | Pseudopapillary structures "Micropapillary arrangement" | May be associated with tumor microenvironment remodeling, indicating increased invasiveness |
| Microscopic Patterns | Trabecular | 7 cases | 32 cases | Trabecular and nested growth "Small beams separated by fibrous stroma" | Suggests invasion along vessels or stroma, often seen in biphasic tumors |
| Microscopic Patterns | Cord-like | - | 28 cases | Thin cord-like arrangement "Anastomosing cord structures" | Common at invasive edges, indicative of poorly differentiated carcinoma |
| Microscopic Patterns | Heterogeneous | 3 cases | - | Mixed growth of undifferentiated small cells and squamous differentiation | Suggests tumor heterogeneity, possibly involving cell populations with varying degrees of differentiation |
| Microscopic Patterns | Whorling | - | 19 cases | Whorled arrangement around vessels "Concentric structures" | Suggests perineural invasion or special differentiation |
| Microscopic Patterns | Cribriform | - | 9 cases | Cribriform pattern "Adenoid cystic carcinoma-like arrangement" | Requires immunohistochemical analysis to rule out adenoid cystic carcinoma |
| Cellular Features | Primitive undifferentiated | 62 cases | - | Primitive epithelial cells "Primitive undifferentiated appearance" | Associated with dedifferentiation caused by NUT gene rearrangement, characterized by BRD4-NUT fusion protein expression |
| Cellular Features | Small-medium size | 53 cases | - | Small to medium-sized (10-15μm) "Medium-sized primitive cells" | Reflects a relatively primitive stage of tumor cell differentiation |
| Cellular Features | Prominent nucleoli | 43 cases | 98 cases | Vesicular nuclei with prominent nucleoli "Cherry-red nucleoli >2μm" | Indicates high proliferative activity (associated with NUT gene rearrangement) |
| Cellular Features | abrupt keratinization | 3 cases | 76 cases | Abrupt keratinization in focal areas "Squamous differentiation without transition" | Diagnostic hallmark of NUT carcinoma, distinguishing it from gradual keratinization in conventional squamous carcinoma |
| Cellular Features | Basaloid | 12 cases | 66 cases | Basaloid cell features "Basaloid cell nests" | Suggests tumor origin from primitive epithelial stem cells |
| Cellular Features | Vesicular nuclei | 21 cases | - | Vesicular chromatin "Nuclei with vesicular appearance" | Indicates active transcriptional state, potentially related to chromatin remodeling by NUT fusion proteins |
| Cellular Features | Monotonous | 29 cases | - | Monotonous cell population "Uniform short spindle cells" | Reflects clonal proliferation of tumor cells |
| Cellular Features | Spindle | 11 cases | 17 cases | Spindle-shaped cells with pointed nuclei "Spindle cells in bundles" | Suggests epithelial-mesenchymal transition (EMT), associated with increased invasiveness |
| Cellular Features | Pleomorphic | 14 cases | 39 cases | Pleomorphic epithelial cells "Cell size variation >3-fold" | Reflects genomic instability |
| Cellular Features | Mitotically active | 24 cases | 48 cases | Frequent mitoses (6/0.24mm²) "Atypical mitoses" | Indicates high tumor proliferation index (Ki-67 often >50%) |
| Cellular Features | Naked nuclei | - | 59 cases | Dispersed naked nuclei "Naked nuclei in apoptotic debris" | Reflects loss of adhesion due to rapid tumor cell proliferation |
| Cellular Features | Apoptotic bodies | - | 32 cases | Starry-sky apoptosis "Eosinophilic apoptotic bodies in cytoplasm" | Indicates hypoxia-induced necrosis caused by rapid tumor growth |
| Cellular Features | Striated | - | 19 cases | Eosinophilic cytoplasmic inclusions "Striated rhabdomyoblast-like features" | May suggest abnormal protein aggregation |

1. Supplementary Table S6. Initial pathologic diagnoses prior to definitive confirmation of thoracic and head-and-neck NUT carcinoma.

| **Type** | **Total (n=229)** | **Thoracic**  **(n=114)** | **Head & Neck**  **(n=115)** |
| --- | --- | --- | --- |
| ****Squamous Cell Carcinoma**** | **84 (36.7%)** | 37 (32.5%) | 47 (40.9%) |
| ****Undifferentiated Tumor**** | **37 (16.2%)** | 17 (14.9%) | 20 (17.4%) |
| ****Poorly Differentiated Carcinoma**** | **34 (14.8%)** | 19 (16.7%) | 15 (13.0%) |
| ****Adenocarcinoma**** | **10 (4.4%)** | 9 (7.9%) | 1 (0.9%) |
| ****Small Round Cell Tumor**** | **4 (1.7%)** | 3 (2.6%) | 1 (0.9%) |
| ****Neuroendocrine Tumor**** | **5 (2.2%)** | 2 (1.8%) | 3 (2.6%) |
| ****Small Cell Carcinoma**** | **5 (2.2%)** | 4 (3.5%) | 1 (0.9%) |
| ****Mucoepidermoid Carcinoma**** | **8 (3.5%)** | 1 (0.9%) | 7 (6.1%) |
| ****Epithelioid Tumor**** | **3 (1.3%)** | 2 (1.8%) | 1 (0.9%) |
| ****Germ Cell Tumor**** | **4 (1.7%)** | 4 (3.5%) | 0 (0.0%) |
| ****Lymphoma**** | **2 (0.9%)** | 1 (0.9%) | 1 (0.9%) |
| ****Basaloid Carcinoma**** | **1 (0.4%)** | 1 (0.9%) | 0 (0.0%) |
| ****Infectious Disease**** | **5 (2.2%)** | 2 (1.8%) | 3 (2.6%) |
| ****Hemangioma**** | **2 (0.9%)** | 0 (0.0%) | 2 (1.7%) |
| ****Adenoid Cystic Carcinoma**** | **1 (0.4%)** | 0 (0.0%) | 1 (0.9%) |
| ****Myoepithelial Carcinoma**** | **1 (0.4%)** | 0 (0.0%) | 1 (0.9%) |
| ****Adenosquamous Carcinoma**** | **2 (0.9%)** | 2 (1.8%) | 0 (0.0%) |
| ****Papillary Carcinoma**** | **2 (0.9%)** | 0 (0.0%) | 2 (1.7%) |

1. Supplementary Table S7. Binary PD-L1 sensitivity summary.

| **Analysis set** | **Three-category result** | **Binary result** | **Test / interpretation** |
| --- | --- | --- | --- |
| Literature-derived PD-L1 subset | Negative 60/78; Low 13/78; High 5/78 | Negative 60/78, 76.9%; Any expression 18/78, 23.1% | Confirms predominant PD-L1 negativity |
| Institutional cohort | Negative 25/33; Low 5/33; harmonized High 3/33 | Negative 25/33, 75.8%; Any expression 8/33, 24.2% | Exact binomial vs literature-derived negativity, *p*=0.838 |
| Fusion + PD-L1 subset | Negative 32/39; Low 5/39; High 2/39 | Negative 32/39, 82.1%; Any expression 7/39, 17.9% | Partner-specific interpretation limited by sparse counts |
| Thoracic squamous vs other strata | Thoracic squamous: Negative 33/37; non-negative 4/37 | Thoracic squamous: 33 negative / 4 any expression; others: 27 negative / 14 any expression | OR=4.28 for PD-L1 negativity; *p*=0.017 |

PD-L1 binary status was defined as Negative versus Any expression. Any expression included cases categorized as Low or High expression in the three-category framework. The literature-derived PD-L1 subset included 78 cases with extractable PD-L1 status. The fusion + PD-L1 subset included 39 literature-derived cases with both definitive fusion-partner annotation and PD-L1 status. The thoracic squamous comparison was derived from the 78 literature-derived PD-L1-assessable cases; thoracic squamous cases were compared with all remaining anatomical–pathologic strata. Institutional cohort percentages were calculated among the 33 PD-L1-tested cases.

1. Supplementary Table S8. Binary PD-L1 status according to fusion partner in literature-derived cases with both fusion and PD-L1 data.

| **Fusion partner** | **PD-L1 Negative** | **Any PD-L1 expression** | **Total** | **Fisher p** |
| --- | --- | --- | --- | --- |
| *BRD4::NUTM1* | 23 | 5 | 28 | 1 |
| *BRD3::NUTM1* | 5 | 0 | 5 | 0.563 |
| *NSD3::NUTM1* | 3 | 1 | 4 | 0.563 |
| *ZNF532::NUTM1* | 1 | 0 | 1 | 1 |
| *ZNF592::NUTM1* | 0 | 1 | 1 | 0.179 |
| Total | 32 | 7 | 39 | — |

PD-L1 binary status was defined as Negative versus Any expression. Low and High expression categories were collapsed into Any expression to reduce misclassification related to heterogeneous assays, TPS/CPS scoring systems, reporting thresholds, and sparse subgroup counts. Fisher’s exact tests compared each fusion partner against all other fusion partners.Owing to sparse cells and zero-event strata, odds ratios were not emphasized, and the results should be interpreted as exploratory.

1. Supplementary Table S9. Key Cell Lines and Their Characteristics in NUT Carcinoma Research.

| **Category** | **Cell line** | **Primary reference**  **(s)** | **Patient / tissue source** | **Fusion type** | **Key characteristics and applications** |
| --- | --- | --- | --- | --- | --- |
| Human-derived | 14169 | Durall et al., 2023 (first mentioned in Grayson et al., 2014) | Patient details not publicly reported; provided by C.A. French | *BRD4::NUTM1* | Standardized human reference line for cross-species comparison with murine models; used for validating BET inhibitor efficacy. In OTTER transcriptomic profiling, classified as mesodermal rather than typical squamous carcinoma, consistent with an extremely low differentiation state. |
|  | TC-797 | Toretsky et al., 2003 | 15-year-old male; mediastinal mass (thymic carcinoma) | *BRD4::NUTM1* | Used to study endogenous fusion protein expression, siRNA knockdown, drug screening, and generation of derivative/engineered lines. |
|  | PER-403 | Kees et al., 1991 | 11-year-old female; intrathoracic undifferentiated carcinoma | *BRD4::NUTM1* | Used for validating knockdown effects, transcriptomic analyses, and drug sensitivity testing. |
|  | HCC2429 | Haruki et al., 2005 | 34-year-old female; lung carcinoma with bone metastasis | *BRD4::NUTM1* | Used to clone breakpoints at 19p13.1 and 15q13.2, confirming fusion transcript architecture. |
|  | Ty-82 | Kuzume et al., 1992 | 22-year-old female; undifferentiated thymic carcinoma | *BRD4::NUTM1* | Early-established line used for cytogenetic karyotyping and nude-mouse xenograft tumorigenicity assays. |
|  | 10-15 (primary culture) | Grayson et al., 2014 | 42-year-old male; pleural effusion | *BRD4::NUTM1* | Primary culture used for ChIP-seq and RNA-seq, enabling discovery/characterization of H3K27ac megadomains. |
|  | Case 1 (Ty-82 related) | French et al., 2001 | 22-year-old female; thymus and respiratory epithelium | *BRD4::NUTM1* | Used in early breakpoint cloning and validation by FISH and Southern blot. |
|  | Case 2 | Not specified | 13-year-old female; epiglottis | *BRD4::NUTM1* | Case-derived material; details not specified in the provided record. |
|  | 10326 | French et al., 2008 | Poorly differentiated carcinoma | *BRD3::NUTM1* | First reported *BRD3::NUTM1* cell line; demonstrated that *BRD3::NUTM1* shares oncogenic logic with *BRD4::NUTM1*, particularly differentiation blockade. |
| Mouse-derived | 311E | Durall et al., 2023 | Tumor from transgenic mouse; gastroesophageal junction (GEJ) | *BRD4::NUTM1* | Retains transcriptomic and epigenetic features of the primary tumor; used for in vivo drug efficacy testing and immune microenvironment studies. 317E serves as an independent biological replicate of 311E. |
|  | 317E | Durall et al., 2023 | Tumor from transgenic mouse; gastroesophageal junction (GEJ) | *BRD4::NUTM1* | Companion murine line to 311E, supporting reproducibility in in vivo and mechanistic studies. |
| Engineered / derivative models | 797TRex | Alekseyenko et al., 2015 | Derived from TC-797 | *BRD4::NUTM1* | Inducible expression model with a tetracycline-regulated system enabling expression of epitope-tagged (FLAG/HA) *BRD4::NUTM1* for protein interaction mapping and subcellular localization studies. |
|  | 797-ZNF532 (BioTAP-tagged) | Shiota et al., 2018 | Derived from TC-797 | *BRD4::NUTM1* (context: ZNF532-tagged complex) | CRISPR-engineered model introducing a BioTAP tag into ZNF532, enabling functional interrogation of the Z4 complex in NUT carcinoma biology. |
|  | –NBioTAP | Not specified | Not specified | *BRD4::NUTM1* (context: ZNF532-tagged complex) | Entry present in source table, but no further details provided. |

1. Supplementary Table S10. In Vivo Animal Model Systems in Basic Research of NUT Carcinoma.

| **Category** | **Model** | **Primary reference** | **Origin / engineering strategy** | **Key features** | **Main applications** |
| --- | --- | --- | --- | --- | --- |
| **Genetically engineered mouse models (GEMMs)** | **NC GEMM (Translocation model)** | Zheng et al., 2024 | **Cre–LoxP–induced chromosomal translocation.** loxP sites were introduced into the mouse Brd4 and Nutm1 loci using CRISPR/Cas9, and Cre recombinase was used to induce a t(2;17) translocation to model the human t(15;19) event. Cre drivers: (1) KRT14-Cre (oral/head-and-neck epithelium); (2) Pdx1-Cre (pancreas); (3) Prrx1-Cre (limb/soft tissue); (4) NLS-Cre (broad systemic expression). | **High fidelity to human NC.** (1) Histology: poorly differentiated squamous cell carcinoma (SCC) with variable keratinization. (2) Molecular features: expression of *BRD4::NUTM1* fusion protein with nuclear speckles; high MYC, p63, SOX2; increased H3K27ac signal. (3) Biology: highly aggressive, rapid growth, and frequent metastasis (lymph nodes, lung, liver). (4) Microenvironment: prominent desmoplasia and epithelial–mesenchymal transition (EMT) features. | **Comprehensive in vivo platform.** (1) Mechanistic studies: oncogenic potency and epigenetic reprogramming across tissue contexts. (2) Metastasis research: notably rapid and widespread metastasis in the pancreatic model. (3) Drug testing: immunocompetent preclinical model for targeted agents and immunotherapies. |
|  | **Sox2-cKO NC GEMM (target validation model)** | Luo et al., 2025 | **Conditional knockout on the NCT background.** The Zheng et al. (2024) translocation model was crossed with Sox2^flox/flox mice; KRT14-Cre or Pdx1-Cre simultaneously induced the fusion event and deleted Sox2 in tumor cells. | **Minimal phenotypic impact.** (1) Penetrance: 100% tumor penetrance despite SOX2 deletion. (2) Pathology/molecular: tumor growth kinetics and poorly differentiated SCC morphology unchanged; *BRD4::NUTM1*, MYC, p63 expression largely unaffected. (3) Transcriptomics: only modest alterations in metabolic pathways; core oncogenic programs preserved. | **Mechanism refinement & target evaluation.** (1) Mechanistic revision: challenges the view that SOX2 is an essential NC driver, supporting strong oncogenic autonomy of *BRD4::NUTM1*. (2) Target assessment: suggests SOX2-directed monotherapy may be ineffective in NC. |
|  | **NC GEMM (Inversion; inducible conditional expression model)** | Durall et al., 2023 | **Cre–LoxP–mediated inversion (FLEx).** An inverted human NUTM1 sequence was knocked into the mouse Brd4 locus; Sox2-CreERT2 (tamoxifen-inducible) activated fusion expression. Tissue context: squamous epithelium of the gastroesophageal junction (GEJ). | **High penetrance with rapid lethality.** (1) Tumorigenesis: 100% penetrance; median survival ~31 days post-induction. (2) Histology: poorly differentiated SCC with high MYC and p63. (3) Epigenetics: formation of H3K27ac-enriched megadomains. (4) Derived cell lines: establishment of murine NC lines 311E and 317E. | **Therapeutic validation & early lesion studies.** (1) In vivo efficacy: BET inhibitor ABBV-744 significantly prolonged survival. (2) Early events: identification of carcinoma in situ (CIS), enabling interrogation of early tumorigenesis. (3) In vitro resources: provides early mouse-derived NC cell lines for mechanistic and pharmacologic studies. |
| **Knockout mouse model** | **Nut-KO** | Shiota et al., 2018 | **Constitutive (whole-body) knockout** of Nutm1. | **Male infertility.** (1) Phenotype: male sterility with late-stage arrest of spermatogenesis. (2) Mechanism: impaired H4 hyperacetylation in spermatids, preventing histone-to-protamine replacement. | **Physiologic function of NUT.** Demonstrates NUT’s normal role in spermatogenesis—recruiting p300 to drive histone hyperacetylation—which mirrors the core oncogenic mechanism hijacked in NC. |
| **Xenograft model** | **Nude mouse xenograft** | Kubonishi et al., 1991 | **Subcutaneous implantation** of patient-derived NC cell lines (e.g., Ty-82) into immunodeficient nude mice. | **Retention of primary tumor features.** Xenografts closely resemble the original tumor histology (undifferentiated carcinoma with t(15;19) rearrangement). | **Early validation platform.** (1) Tumorigenicity testing: confirms malignant potential of cultured cell lines in vivo. (2) Drug screening: used for early in vivo evaluation of agents such as BET inhibitors. |

1. Supplementary Figure S1.Research Impact by Department: Total Citations, H-index, and Average Citations per Publication


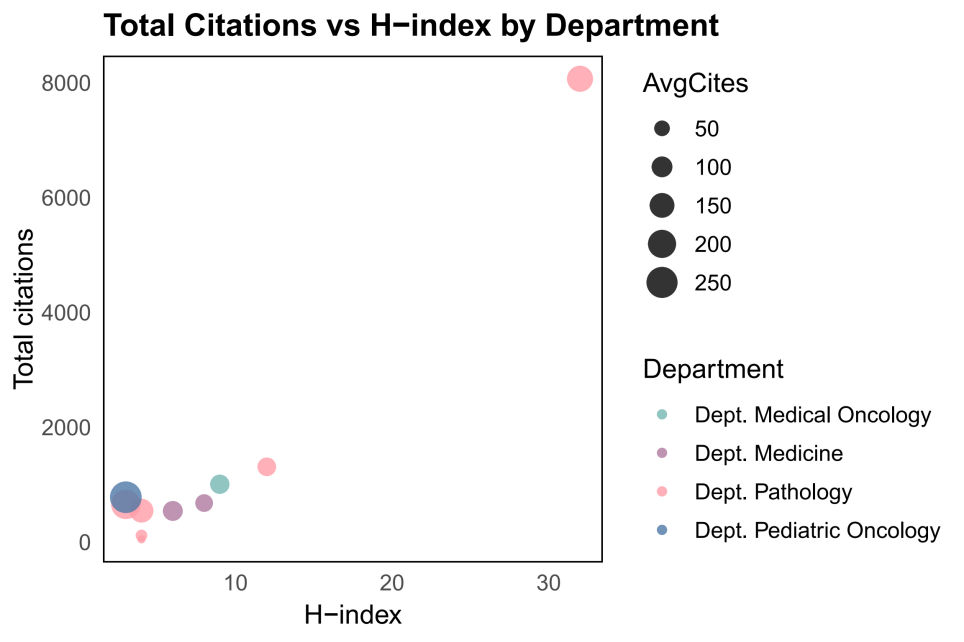


1. Supplementary Figure S2.Temporal Citation Heatmap of the Top 10 Most-Cited Publications on NUT Carcinoma


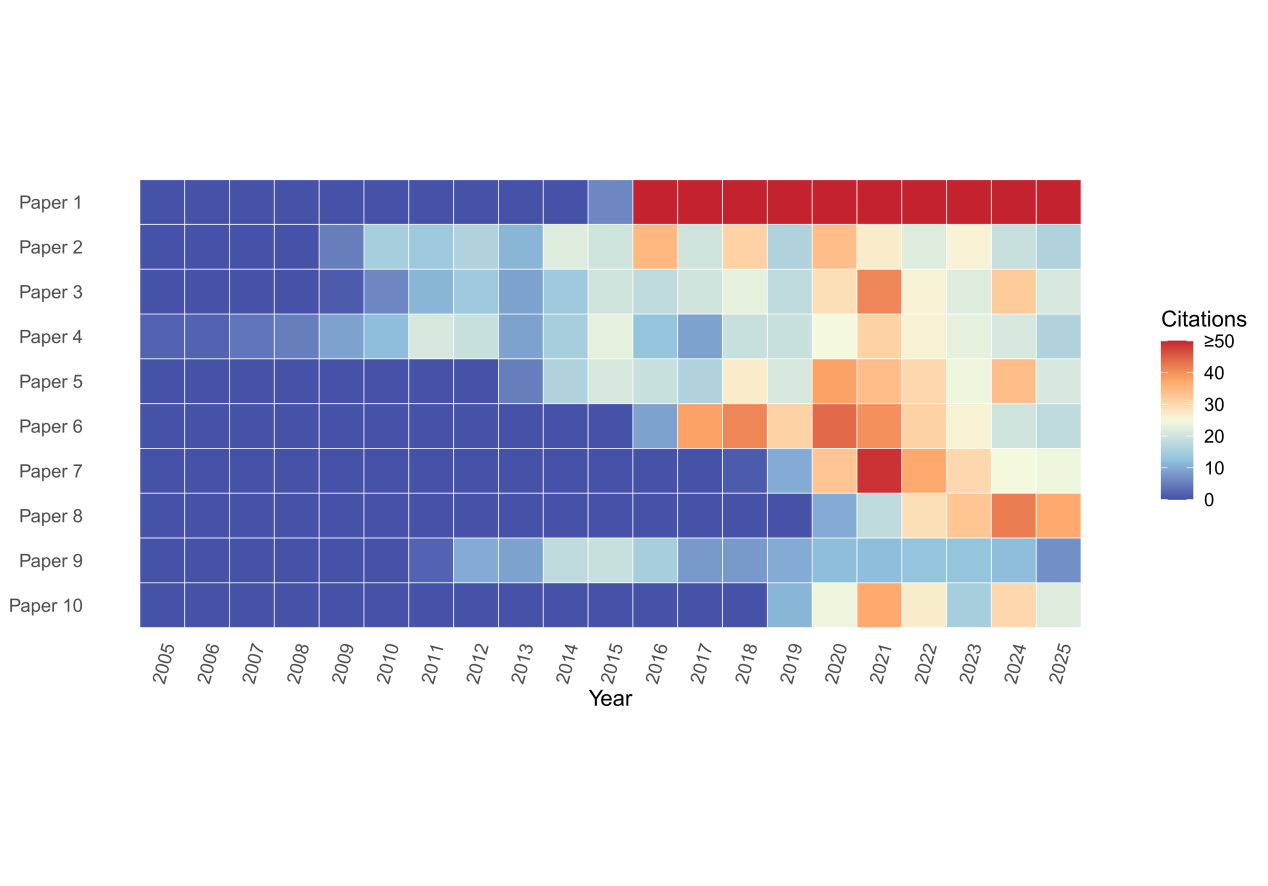


The heatmap illustrates annual citation patterns for the 10 most-cited publications from 2005 to 2025. Color intensity represents citation frequency (0 to ≥50 citations per year). Sustained citation accumulation is evident after 2015, with pronounced citation bursts during 2018–2025. Key publications include: Paper 1 - Travis et al. (2015) "The 2015 World Health Organization Classification of Lung Tumors: Impact of Genetic, Clinical and Radiologic Advances Since the 2004 Classification" published in Journal of Thoracic Oncology (3,613 total citations, peak: 556 in 2021); Paper 2 - French et al. (2008) "BRD-NUT oncoproteins: a family of closely related nuclear proteins that block epithelial differentiation and maintain the growth of carcinoma cells" in Oncogene (349 citations); Paper 3 - Haack et al. (2009) "Diagnosis of NUT Midline Carcinoma Using a NUT-specific Monoclonal Antibody" in American Journal of Surgical Pathology (325 citations); Paper 4 - French et al. (2004) "Midline carcinoma of children and young adults with NUT rearrangement" in Journal of Clinical Oncology (323 citations); Paper 5 - Bauer et al. (2012) "Clinicopathologic Features and Long-term Outcomes of NUT Midline Carcinoma" in Clinical Cancer Research (306 citations); Paper 6 - Stathis et al. (2016) "Clinical Response of Carcinomas Harboring the BRD4-NUT Oncoprotein to the Targeted Bromodomain Inhibitor OTX015/MK-8628" in Cancer Discovery (298 citations); Paper 7 - Lewin et al. (2018) "Phase Ib Trial With Birabresib, a Small-Molecule Inhibitor of Bromodomain and Extraterminal Proteins, in Patients With Selected Advanced Solid Tumors" in Journal of Clinical Oncology (211 citations); Paper 8 - Chau et al. (2020) "An Anatomical Site and Genetic-Based Prognostic Model for Patients With Nuclear Protein in Testis (NUT) Midline Carcinoma: Analysis of 124 Patients" in JNCI Cancer Spectrum (169 citations); Paper 9 - Schwartz et al. (2011) "Differentiation of NUT Midline Carcinoma by Epigenomic Reprogramming" in Cancer Research (168 citations); Paper 10 - French (2018) "NUT Carcinoma: Clinicopathologic features, pathogenesis, and treatment" in Pathology International (166 citations).

1. Supplementary Figure S3.Co-citation Network of Publications in NUT Carcinoma Research


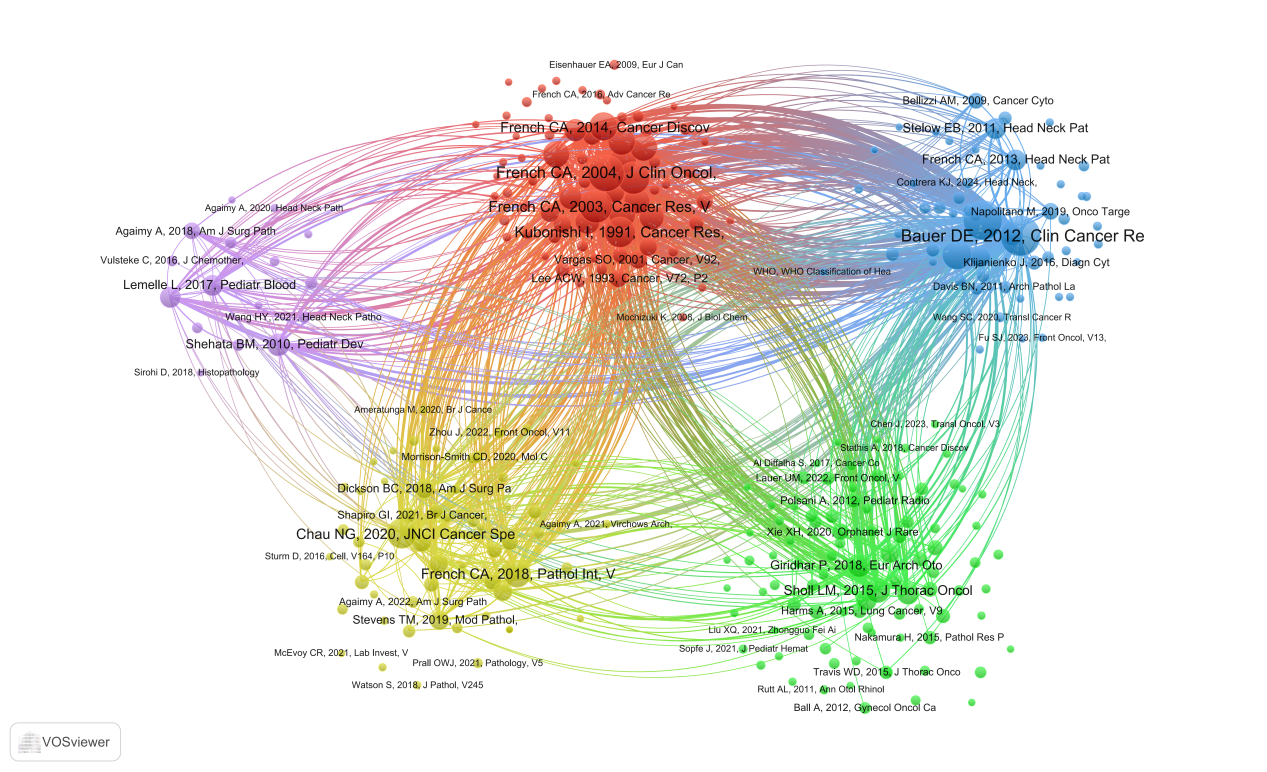


1. Supplementary Figure S4.Timeline Visualization of Keyword Evolution in NUT Carcinoma Research


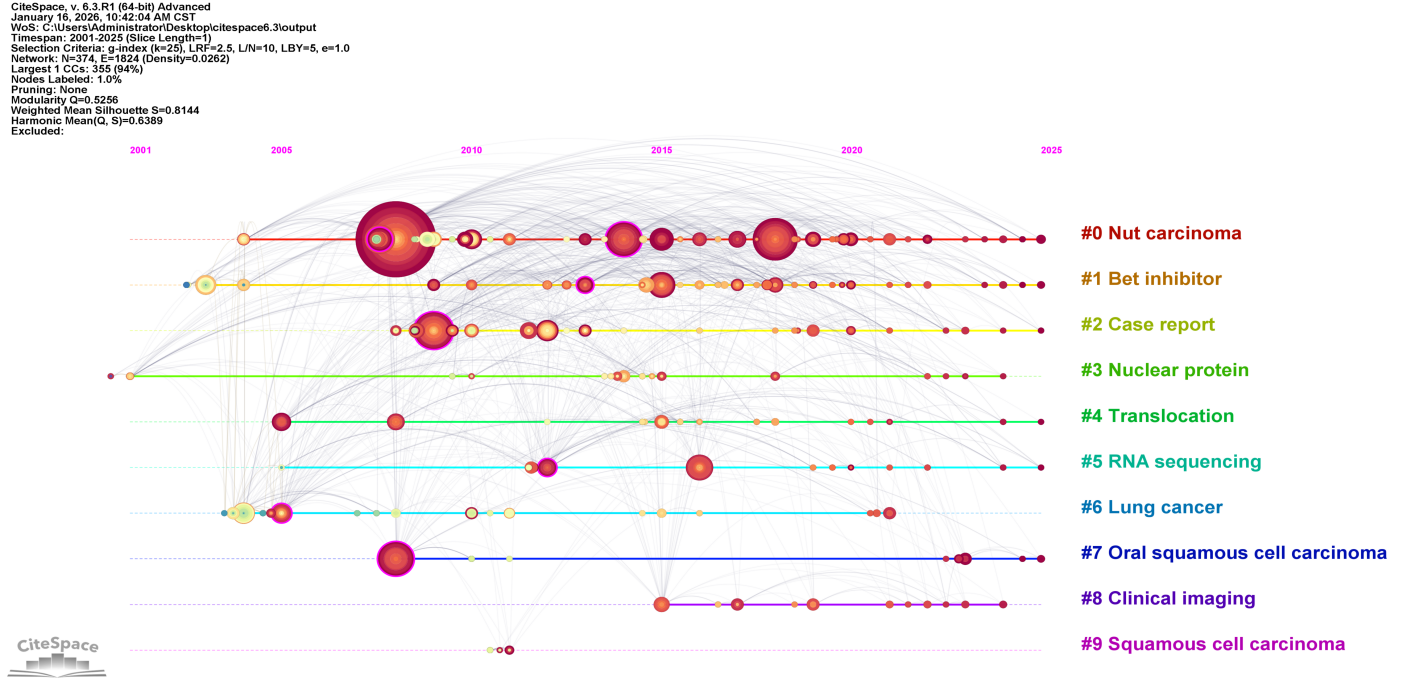


1. Supplementary Figure S5. Enrichment analysis (OR with 95% CI; Fisher’s exact test) for fusion partner–stratum associations (e.g., BRD4::NUTM1 in squamous/thoracic-squamous; YAP1::NUTM1 and MGA::NUTM1 in selected non-squamous strata)


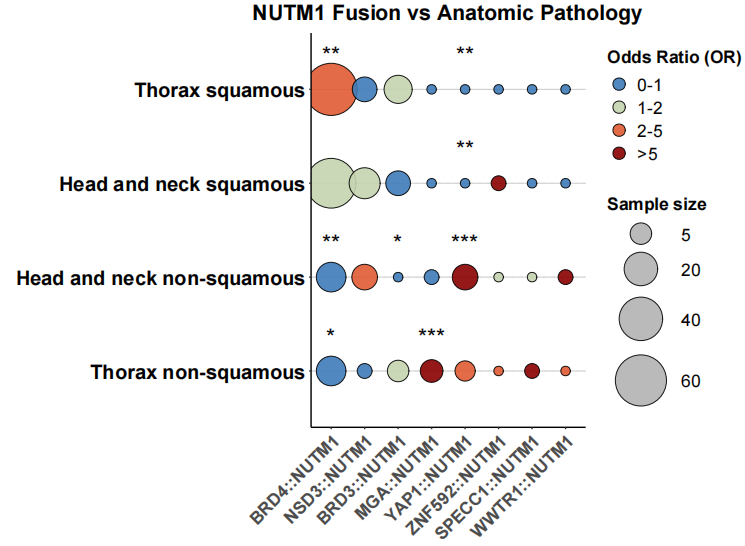


1. Supplementary Figure S6.Stratified frequency visualisation of fusion partners across predefined anatomical–lineage strata


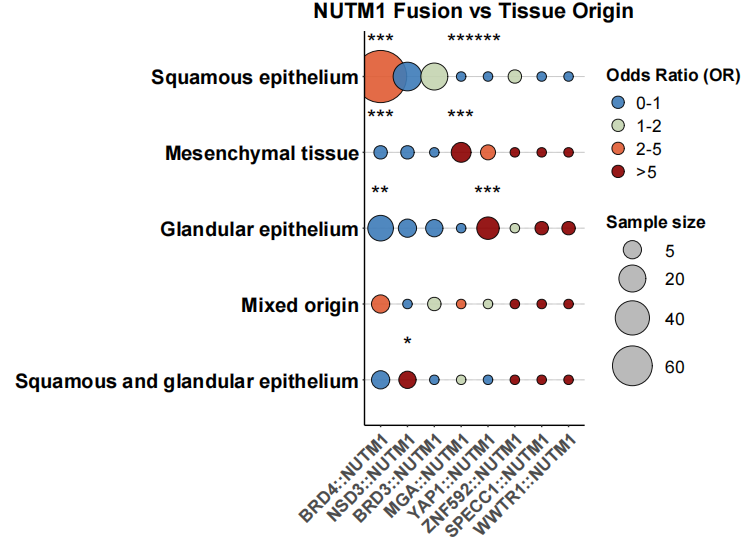

Supplement: Supplementary file 1 [file Supplementaryfile1.docx]
